# Extra-Limites

**Published:** 1827-04

**Authors:** 


### EXTRA-LIMITES.

### PRIZE HOSPITAL REPORT,

### No. I.

MR. THOS. H. SMITH, PUPIL,

ST. THOMAS'S HOSPITAL, BOROUGH.

I.

TWO CASES OF INJURY OF THE SPINE, TREATED BY F. TYRRELL, ESQ. AT ST. THOMAS'S HOSPITAL.

1. *Case of Fracture of the Spine, with displacement; with the Operation, &c.*

| | |
|---|---|
| JEREMIAH MAHONY, Æt. 26 Yrs. Treated by Mr. TYRRELL. | Isaac's Ward, St. Thomas's Hospital. |

1827,

*February 5th.* J. M. was brought to the hospital, about 2 o'clock, P. M. on 5th Feb. with the account, that an immense piece of timber (said to weigh near 2 tons) had fallen on his back.

There was paralysis of the lower extremities, with tension, and considerable effusion in the region of the loins; there was also loss of sensation from the tuberosities of the ischia, and, on more minute examination, by Mr. Tyrrell, it appeared evident that there was displacement of the (?) last dorsal or first lumbar vertebra, He was a strong and well made man, and evidently of a good constitution. His pulse was 70 and strong—his feet were at first cold, but soon became warmer. Mr. T. ordered his dresser, Mr. Whitworth, to apply leeches—fomentation, and afterwards spirit-wash during the night, so as to relieve the tension and effusion as much as possible. The catheter was introduced, he having, of course, lost all power of voiding his urine. *February 6th.* From the perseveran[illegible] of the attendants in the plan ordered last evening, the effusion was considerably lessened, and the depression of the spinous processes could now be much more distinctly felt; therefore, Mr. T. could now have not the slightest hesitation as to the plan to be adopted; he having determined, although his former case terminated unsuccessfully, to repeat the operation of removing the arch of the vertebra, which pressed the medulla spinalis, whenever a case favourable for it should present itself; and such the case under consideration appeared to be. Mr. T. therefore, now sent for his able colleague, Mr. Green, in order to have the advantage of his countenance and advice. The pulse was 70 and full—he had had no sleep—complained of much pain in his back, but none elsewhere—feet warm—bowels not yet open.

*Operation, by Mr. Tyrrell.*

The patient being placed in as favourable a position as possible, with his chest somewhat raised, so as to have the light full on the back, an incision was made over the spinous processes, to the extent of five or six inches, the depressed vertebra being in the centre of the incision. The muscles were then dissected off between the spinous and transverse process of the vertebra above, and the one below, as well as from that which was displaced, so as to expose completely the arches. It was found very useful in this, as in Mr. T.'s former operation, to have two bent pieces of steel, with which the assistants could very satisfactorily keep apart the divided muscles, and bring every stage of the operation within the view of the operator. Having thus exposed the arches of the vertebræ, it was then found that the *inferior* articular processes of the 12th dorsal were thrown *for-*

*wards* before the superior of the first lumbar; consequently, the arch of the 12th dorsal was brought forwards, and pressing the medulla spinalis, having the superior articular process of the first lumbar vertebra in contact with the inferior part of it.

Mr. T. then proceeded with a chain-saw to take off the spinous process of the 12th dorsal, that he might the more clearly obtain a view of the arch, which it was next his object to remove. In order to accomplish this, Mr. Tyrrell, in his former operation, at first tried a small trephine, as recommended, and, indeed, used by Mr. Cline; but, finding this not to succeed, he was, in that case, under the necessity of finishing the operation with a Hey's saw, which he found much better adapted for the operation. This instrument was, therefore, again used, and the arch was sawn through as near as possible to the transverse processes. To thus say "sawn through," would, perhaps, lead to the idea, without further explanation, that it was *at once* divided with impunity; but such was not the case; for so closely was the arch in contact with the dura mater of the spinal cord, as to render it necessary to be extremely cautious; and when a part was sawn through, it was removed by a strong pair of curved forceps: this was repeated several times before the spinal chord was exposed, which was ultimately done without injuring the membrane, which was found entire, without any appearance of effusion: and, as far as could be ascertained by its external appearance, the chord was uninjured. It having been ascertained, by the introduction of a probe, that no pressure remained, the wound was carefully cleaned, and the edges brought together, or nearly so, by a plaster, which Mr. Tyrrell uses, composed of emp. thur. c. sapone, covered with simple dressing. The operation lasted less than an hour. Very little blood was lost, and the patient bore it with the greatest fortitude.

He was conveyed to bed, and placed upon his side, rather inclining, however, on his face.

*Vesp.* Pulse 90 and full—*sensation lower than before the operation.* An elastic gum catheter was introduced, and ordered to be worn in the bladder, by means of which the attendants could, as often as needful, draw off the urine.

*7th.* Sensation is now about nine inches lower than before the operation—bowels not yet open—pulse 90 and full—has not slept—has some thirst—oozing of a bloody serum from the wound—urine drawn off several times, is plentifully secreted, and natural. The lower extremities are of same temperature as the rest of the body. Ordered enema c. dect. hord, c. sod. mur. R. Ol. ricin. ℥ss. statim et post horas 4, si opus sit. Finally, levigated calamine powder, was ordered to be dusted on the scrotum and in the perineum, to prevent excoriation.

*8th.* Has slept several hours in the night—the bowels are not open—pulse 94, more full and sharp—appears very cheerful and confident—wound is suppurating—*the sensation is not so low in the extremities as yesterday.*

*Ordered.* Enema colocynth. Statim, et post hor. vj. si opus sit.

*Vesp.* Both the clysters had been given, and the bowels have been relieved. In order to keep up the action, the following draught was ordered to be given occasionally. R. Mag. sulph. ʒij. aq. menth. p. ℥iss. Urine plentiful and natural—extremities continue warm—a little slough on the surface of the wound—complains of rather more pain in and surrounding it—has also a slight pain in the head—pulse as in the morning.

*9th.* The bowels continue open—has taken the draught three times—he continues in good spirits—has some appetite—has been hitherto allowed arrow-root, sago, and other light food—prickling sensation in the legs—the urine is very plentiful, and now, for the first time, smelt slightly ammoniacal. Pulse 95 and full.

*Vesp.* The prickling sensation continues in the extremities, and the urine evolves rather more ammoniacal odour than in the morning—pulse 100, but not hard—pain in the head—sensation, on pinching the extremities, not lower than yesterday. The bladder was injected with lukewarm water, which was allowed to pass away immediately; about ½ pint more being then injected, and suffered to remain in till it became again necessary, from the accumulation of the urine, to allow it to pass off. This was ordered to be repeated several times a day. The wound appeared, on dressing it this evening, more healthy than yesterday.

*10th.* His bowels continue open—has some pain in his head—he continues cheerful—his tongue this morning, for the first time, furred—his pulse is not so full as yesterday, and 80—his skin is rather dry, and he feels, he says, slightly feverish—there is a free discharge from the wound. Mr. T. considered it advisable to increase the nutrition of his diet, which had hitherto consisted of, as I have before observed, light pudding, sago, arrow-root, &c. He, therefore, ordered him to have daily a beef-steak or mutton chop; this the patient appeared gratified to hear. He took the former to day with a relish: he had also ½ pint of porter; but this, it was found, stimulated him too much, and he afterwards had the table beer of the hospital. He was ordered a fever draught to take occasionally—his urine had been drawn off twice in the night (pint and ½ each time)—it was also drawn off this morning and the bladder injected with warm water as before. The urine was more ammoniacal, and was mixed with a small quantity of thick mucus—there was also a very small portion of blood—continues to have the "darting and prickling" feel (as he describes it) in his legs and thighs —sensation is not quite so low down as last evening.

*Vesp.* His bowels have not been open since the morning—complains of considerable pain in the wound—sensation is not lower than the crista of the ilium —his pulse more full than in the morning —he also complains of soreness in the chest, and some difficulty of breathing from his position. Mr Tyrrell in consequence changed it, placing him on his back with a firm pillow on each side of the wound in the direction of the spine, so as to prevent pressure. He expressed himself very warmly as to the immediate relief this afforded him in his breathing, &c. but as he still complained of soreness, he was examined, and a slight redness was perceived in the part. His aperient draught was repeated. The surface of the wound was covered with a slough.

*11th.* Has slept well—feels more easy in his present position—pulse 80 and full —has not so much pain in the head—has still pain in the back, though not so violent as yesterday—tongue white—bowels freely open—has more of the prickling in his legs and thighs—urine plentiful—still ammoniacal—bladder injected with water —more mucus mixed with it, but not any blood.

*Merid.* Enjoyed his mutton chop—continues to take plentifully of arrow root, &c.

*Vesp.* Pulse 90, rather harder than in the morning—bowels open—rather more restless—dull pain in the bowels, with some soreness of the integuments—his breathing appears a little more hurried than natural—wound still looks rather foul—sensation is not lower than yesterday—there is more mucus mixed with a little blood in the urine this evening—it also emits a stronger ammoniacal odour—ordered to continue the fever mixture and to have tinct. opii gtt. xx. hora somni. Bladder injected as usual.

*12th.* Slept for a few hours last night, but became more restless towards morning—complains of more pain in the bowels which are open—his pulse is as last evening, and his countenance had not the placid appearance it had hitherto borne—there was an expression of pain in it—there was some pain (he did not however complain much of it) in his chest, on the right side, where he had complained of pressure in his first position—sensation is not so low by six inches as yesterday—there is no darting in the legs and thighs nor any pain in the head. The urine is strongly ammoniacal, and has more thick mucus (with a little blood) than yesterday—there is not much pain in the wound—the slough is separated and it looks more healthy.

*Vesp.* Urine is plentiful—on the bladder being injected with warm water, much mucus came away—it also smelt very ammoniacal—bowels open—fæces dark coloured—his countenance expresses more anxiety, and he is not so confident as to his recovery, as he has hitherto been —has still slight pain in the chest—pulse 90, sharper than in the morning—wound dressed looks as well as in the morning—sensation is not lower than the umbilicus.

*13th.* There is this morning an evident alteration in his appearance—his countenance expresses considerable anxiety—has not slept—pulse 100 and hard —has considerable pain in right side of the chest, with cough, which increases the pain—bowels open—urine plentiful—having, as before, ammonia—thick mucus and some blood—the wound looks more unhealthy than last evening, having a slough on the surface—sensation not lower than last evening.

*Ordered.*—V. S. ad vj. vel viij. ozs. and if this does not afford relief, 12 leeches to be applied to the seat of pain. The meat and beer are to be omitted, but to be still allowed sago, arrow-root, or broth.

R. Calomel, gr. j. opii, gr. ss. nocte et mane.

R. Sodæ subcarbonat, ʒiij. mucil. g. acaciæ, ℥ss.

R. Tinct. hyosciam. ʒj. syr. papav. alb. ℥j. aq. menth. ℥vj. M. The patient was directed to take a dessert spoonful of this when the cough was troublesome.

*Merid.* ℥ix. of blood were taken, which presents a buffy appearance—immediately after the bleeding, the pulse became more expanded and soft, but the pain and difficulty of breathing not being relieved, the 12 leeches were applied, which are now bleeding very freely, but his breathing is still laborious—his pulse is 100, though not so hard as in the morning—bowels open—urine drawn and bladder injected. Continues very restless, and his temper which has, till now, been comparatively mild, is very irritable.

10 *o'Clock P.M.* The leeches have continued to bleed very freely—but all his symptoms have now assumed a most alarming aspect. His pulse is 120, full and bounding—has excruciating pain in the side—much greater anxiety of the countenance—great restlessness—frequent cough, with greater difficulty of breathing—pain in the wound, which looked unhealthy—no sensation below the displaced vertebræ. A much greater evolution of ammoniacal odour from the urine, which contained at least 3 ozs. of mucus mixed with blood.

V. Sectio ad ℥xvj. this had a considerable effect for a time on his pulse, but did not relieve the difficulty of breathing, and but very slightly the pain.

2 *o'Clock A. M.* The pulse has risen to 130, but more feeble—pain and difficulty of breathing much more considerable.

V. S. ad ℥vj. produced but temporary relief, and at 4 o'clock A. M. he died.

He was perfectly sensible till within a short time of his death, when he became delirious, and expressed a wish to get out of bed, but did not attempt it. I mention this as a curious coincidence with Mr. Tyrrell's former case, and also in confirmation of Mr. Bell's statement.

It is to be regretted that no post mortem examination could be obtained, as the poor man's Irish friends interfered, and nothing could induce them to consent to it—application was made to the Coroner for an order, but without effect.

*Remarks.* Although this case has terminated unfavourably, still, on looking to the immediate cause of death, which, as far as the symptoms inform us, without having the confirmation of a post mortem inspection, was evidently seated in the pleura and lungs; where, it appeared, on enquiry, he had before had an inflammatory attack; we must acknowledge, that as this attack was not a *necessary* consequence either of the accident or treatment, (which is proved by Mr. Tyrrell's former case) it affords, in this point of view, no argument against the mode adopted for the relief of the patient. But it becomes a question whether it is not almost certain, from the symptoms, that death must have eventually, and at no distant period, terminated the case, from the disorganization evidently going on in the bladder, if the fatal attack of pneumonia had not supervened?—Mr. Tyrrell has, in his remarks on his former case in Sir A. Cooper's Lectures, Vol. 2nd. stated "My patient died of inflammation of the bladder, *occasioned by the irritation of the urine,* which, I believe, *might have been prevented;* and I should have taken steps for that purpose, had I known some circumstances connected with Mr. Cline's experiments relative to injuries of the spine." "He invariably found, that, after complete paraplegia was produced by the injury which he inflicted on the spinal marrow of dogs—the bladder became affected from the *action of the urine on its mucous coat.*" "*This organ,*" he continues, "*having lost its nervous power,* it appears that the *urine becomes decomposed in it, as it does after it is voided under ordinary circumstances,* and thus acts as an *irritant* on the *mucous surface.*" "This" says Mr. T. "might probably be *obviated* by frequently *emptying the bladder* with a syringe, and *injecting a fluid* to protect its mucous coat."

It is, however, necessary that these changes should be further investigated: the present case entirely disproving the above conclusions—1st, "That the urine is decomposed *in the bladder*"—2dly, That it is this, which, acting as an irritant on the mucous coat, produces the mischief; and, lastly, That the diorganization may be "obviated" by the means recommended.

For, in the first place, the urine was drawn off so frequently in the present case, as not to allow sufficient time for the decomposition; but, to place the matter beyond a doubt—the following method was adopted:—After the bladder had been emptied of the urine, by means of Read's syringe, (which is admirably adapted to the purpose) warm water was several times injected and withdrawn, till no trace of ammonia could be perceived. A small quantity of clear water was then injected, and allowed to remain about 15 or 20 minutes, when it was allowed to pass off, and the *odour of ammonia could be distinctly perceived.* Now, supposing urine to have been instantly mixed with the water after its injection into the bladder, and that under the "loss of nervous power," it will decompose as quick in as out of it; still, every one is aware, that more time, than was allowed, is required, under "ordinary circumstances," out of the bladder, for its decomposition.

It must, therefore, be concluded, that in whatever way the ammonia is formed, it takes place before the urine arrives in the bladder.

2ndly. The same argument is equally applicable to this position, the urine not having probably in this case been allowed to remain sufficiently long to produce the disorganization—but

3rdly. In whatever way the disorganization is effected, it is very evident that the means recommended and employed in this case, are perfectly inadequate to the prevention of it; and, as this appears a sine qua non in the successful treatment of these accidents, it still remains an interesting subject for investigation; for it is evident, from the symptoms, that the present case must, ere long, have terminated fatally from the disorganization in the bladder (which it will be seen gradually increased from the 3d or 4th day up to his death) if the fatal attack of pneumonia had not supervened.

An occasional instance may, however, occur where this is not the case. This is exemplified by an interesting case which has within the last few weeks been spoken of at the Borough schools. In this instance, the patient survived a fracture with displacement of the superior lumbar vertebræ, causing paralysis of the lower extremity, 14 years; it was found, on inspecting the body after death, that the spinal chord was completely divided, with the exception of the dura mater. The case is, however, I am told, shortly to be published.

2. *Case of Injury to the Spine.*

W. Stephens, æt. 24, was admitted into St. Thomas's Hospital, under Mr. Tyrrel, on the same day as the case before related, Feb. 5th, with total loss of motion and sensation in the lower extremities, and partial loss of motion in the upper, occasioned by being violently thrown, in a scuffle, against the edge of a board, on his back. There was considerable pain between the shoulders, extending down his arms, with some pain in the head. After minute examination, no irregularity could be perceived in the spinous process. Mr. Tyrrell did not, therefore, consider himself justified in proposing an operation. His pulse was 80, and full—ordered to be cupped to 16 ounces, at the back of the neck, after which, a dose of castor oil. The catheter to be introduced.

*Vesp.* The pain was somewhat relieved by the bleeding.

*Feb. 6th.* Restless night—countenance rather anxious—pulse 85, full—some pain in the head—tongue rather furred, with some thirst—fæces pass involuntarily.

*Vesp.* Urine plentifully secreted, did not smell ammoniacal—pulse 95, rather sharp—more pain and restlessness—cupping repeated, which again produced temporary relief; the pulse also became softer.

*7th, Mane.* Slept for a short time after the bleeding last evening, but has since been restless—the lower part of the abdomen tense, somewhat tender on pressure, and slightly distended—the urine was plentifully secreted—did not smell ammoniacal—there was more anxiety of countenance, and greater difficulty of breathing than yesterday:—the latter symptom was, indeed, now the most urgent; it was, therefore, considered probable, that the injury extended to the cervical vertebræ—his pulse was more feeble.

*Vesp.* Countenance livid, and very anxious—pulse more feeble—bowels evacuated.

*8th.* Was in every respect worse—pulse more feeble—respiration very difficult—anxiety of countenance and restlessness much increased—urine smelt slightly ammoniacal—no mucus mixed with it. He gradually sunk during the day, and in the evening expired.

*Sectio Cadaveris.* Before removing the arches of the vertebræ, after having made an incision upon them, considerable effusion of blood was found, and slight displacement of the 7th cervical vertebra forward was perceptible. Having removed the arches, considerable effusion of a bloody serum was found between the pia and dura mater—extending principally from the 5th cervical to the 4th dorsal vertebra. When the spinal cord was removed and examined more accurately, it was found that at the part opposite to the displaced vertebra, it presented a pulpy, homogeneous mass, containing many bloody points. The pia mater was turgid with blood.

The remaining parts of the vertebræ, at the seat of injury, were then removed, when it was found that the 7th cervical was dislocated forward, with fracture of the left inferior oblique process, the right inferior process being dislocated from the corresponding one on the 1st dorsal vertebra—the intervertebral substance between the 7th cervical and 1st dorsal was torn through transversely—the anterior part adherent to the vertebra above, the posterior to that below—there was also a longitudinal fracture through the body of the 3rd dorsal vertebra.

The cavities were not examined.

---

### II.

### CASE OF VENEREAL LEPRA, FOLLOWING GONORRHŒA AND SUPERFICIAL ULCERATION. TREATED BY F. TYRRELL, ESQ. ST. THOMAS'S HOSPITAL.

JAMES BIRBECK, æt. 20, was admitted into Lazarus' ward, Feb. 1st, 1827. Had gonorrhœa 8 months since, of which he soon recovered; after which, he had superficial ulcers on the glans, which healed in a few days without mercury—took merely six aperient pills.

About a month since, he first found his throat slightly sore, and at the same time had an eruption over the whole body, except the face; it came out at first, he states, "with an itching." Had never any eruption before.

*Feb. 1st.* There is now superficial ulceration on the tonsils, the right being rather the worst—the eruption is in distinct red patches, somewhat elevated, and covered with scales—if these scales are rubbed off, they will form again in a few hours—on the scale being removed, the red base is rather tender, but no appearance of ulceration. He has also a slight excoriation on the glans-penis—has been under medical treatment three weeks before his admission—taking sulphur, and anointing the eruption with sulph. ointment, but without any benefit.

*Ordered.*—Pil. Plummer. gr. v. om. nocte—decoct. sarsæ, c. ℥xij. in die—mist. aper. pro re natâ.

Under this plan the excoriations on the penis were removed in two days, and the superficial ulcers on the tonsils were healed in four or five. The scales also separated in about the same period, and they did not re-form, leaving the red patches, which, however, soon became level with the surrounding skin; by continuing the medicine, the eruption became gradually paler, and on the 5th of March they are scarcely visible.

He is to continue the medicine a few days. Not the slightest effect has ever been produced on his gums.

---

### III.

### CASES OF INTERMITTENT FEVER.

DR. BROWN, in his work on Intermittent Fever, has stated that "the practice of waiting for the paroxysm, and exercising powers to arrest its progress on its first approach, has never been established." Now, as far as the recommendation of a respectable physician, founded on and supported by cases, which were related, illustrating the efficacy of this practice, can be said to establish it, Dr. B.'s assertion is disproved; I allude to the recommendation, by Dr. Ridgway, of this plan, in the Number of the Medical and Physical Journal, for April, 1825.

His attention was first directed to this mode of administering the bark, by a patient taking, at one dose, 1½ oz. instead of in divided ones, as ordered: it prevented the expected fit, and produced nothing unpleasant. "Since this period," says Dr. R. "I have never thought of giving the Peruvian bark in divided doses between the paroxysms; but, where I thought it necessary to use this medicine, have uniformly ordered it in one efficient dose, as *near as possible to the approaching paroxysm.*" Dr. R. adds, that he considers an ounce as a sufficient dose in most instances, to avert the paroxysm—this being accomplished, half-ounce doses were afterwards given. Dr. R. twice cured himself in this manner.

Thus we see the principle of Dr. Brown's practice has been before recommended, but he has had the advantage of possessing the bark in a much more convenient form for fulfilling his object; this preparation having, since Dr. Ridgway first adopted the plan under consideration, been brought more under the notice of the profession; and, in availing himself of it, Dr. B. has, in some measure, altered the mode of fulfilling the object which both have in view, viz. preventing or "repelling," as Dr. B. calls it, the approaching paroxysm, by giving several smaller, in lieu of one efficient dose, immediately before the paroxysm; but still it will be seen the principle is the same. Whether this alteration in the mode of administering the bark is an improvement, on the one recommended by Dr. R. remains to be proved. The following are, however, the results of several cases treated in St. Thomas's Hospital, by Drs. Williams and Roots, they having, with their usual kindness, consented to try the experiment.

It will be seen that only half the dose recommended by Dr. Ridgway was found necessary.

*Case* 1. *Treated by Dr. Williams.*
Wm. Cuff, æt. 35.

*Feb.* 8*th.* Had intermittent fever since Christmas—type quotidian. Bowels regular—slight pain and fulness in left side.

*Ordered.* R. Pulv. cinchon. ℥ss. p. zingiber. gr. xxv. ℳ.

This was ordered to be taken about an hour before the expected paroxysm. Attack not so severe, and much shorter than it had hitherto been—had not, however, taken the bark more than five minutes before the fit came on. Bark did not produce any sickness.

9*th.* Took the dose as yesterday—no attack.

10*th.* Pergat.—no return of paroxysm.

25*th.* Has not had any return of the paroxysm. Continued well up to the 1st of March, when he was discharged.

*Case* 2. Thos. Olives, æt. 36, *from Sheerness. Under Dr. Williams.*

*Feb.* 8*th.* Had ague six months—severe cough, does not expectorate—no pain in chest—bowels lax—pulse 80—type, quotidian.

*Ordered.* R. Pulv. cinchon. ℥ss. p. zing. gr. xxv. Took it an hour before the paroxysm came on; it was very slight. No sickness from bark.

9*th.* Pergat—very slight chill to day.

25*th.* Not any return of the paroxysms.

*March* 1*st.* Discharged, cured.

*Case* 3.—Timothy Flynn, Æt. 27.

*Feb.* 8*th.* Had ague three weeks—health otherwise good—type quotidian. The dose as in former cases—felt but a slight trembling.

9*th.* Pergat—scarcely any cold feel.

10*th.* No attack. 11*th.* The medicine omitted this morning, and he has had an attack of chill.

12*th.* Pergat—no attack. 13*th.* *Rejected the medicine,* and had a more severe attack than the last.

25*th.* Has not had any symptom of ague since.

*March* 1*st.* Discharged, cured.

In the following case the old plan was adopted, to contrast the effects.

*Case* 4.—John Kelley, Æt. 30.

*Feb* 8*th.* Has had intermittent fever two months, daily—bowels regular—some cough at night—pulse 75.

R. Pulv. cinchon. ʒj. 4tis horis, sumend. inter paroxys. opii, gr. j. om. nocte.

8*th.* Shook 1 hour and a half. 9*th.* Pergat—paroxysm came on three hours later, and cold stage lasted an hour.

10*th.* Pergat—same as yesterday.

11*th.* Pergat—continues the same.

12*th.* Somewhat better—pergat.

16*th.* Had an attack every day, but not so violent—pergat.

19*th.* Is still better—pergat. 25*th.* Has a very slight trembling—pergat—he continued the bark for another week, and was discharged cured.

The following were under Dr. Roots.

*Case* 5.—Mrs. A——, ÆT. 30.

*Feb.* 22*d.* Intermittent fever 6 months, daily.

℞. Sulph. quinin. gr. v. acid sulph. gtt. vj. aq. ℥j.

Took it an hour before the expected paroxysm, but it did not occur.

23*d.* *Took no medicine to day*, but the paroxysm *did not return.*

Dr. Roots being thus satisfied of the power possessed by a dose of quinine, thus administered, of preventing the paroxysms, still thought it advisable, in this and in the following cases, to order small doses to be repeated for a few days; the advantage of the practice appearing to consist in making a sudden impression on the constitution; but which impression subsiding, the paroxysms *might* return, to prevent which, this simple precaution only appears necessary. Sulph. quin. gr. iij. ter die, were, therefore, ordered to be taken for a week.

*Case* 6.—SAMUEL ELLIOT, ÆT. 25. Quartan ague six months.

*Feb.* 22*d.* ℞. Sulph. quinini, gr. v. pro dosi.

Is to take one dose about an hour before the expected attack, and, if shivering comes on, to repeat it.

23*d.* Took but one dose—had no attack.

24*th.* Sulph. quin. gr. iij. ter die sumend.

*March* 1*st.* No return.

*Case* 7.—JOHN M'GRAFF, ÆT. 30. Quotidian ague 2 months.

*Feb.* 22*d.* ℞. Sulph. quinini, gr. v. pro dosi.

Took one dose before the expected paroxysm; it came on as usual.

23*d.* Took two doses, and it did not come on.

24*th.* Pergat. No attack to day.

25*th.* Sulph. quinin. gr. iij. ter die sumend.

*March* 1*st.* No return.

*Case* 8.—JEREMIAH ABBOTT, ÆT. 35. Quotidian ague 6 months.

*Feb.* 22*d.*—P. Quinini, gr. x.

To be taken a near as possible to the paroxysm—slight shivering only to day.

23*d.* Pergat—no attack to day.

24*th.* *Did not take any medicine. Had no return.*

*Ordered.*—Sulph. quinini, gr. iij. ter die sumend.

*March* 2*d.* No return.

*Case* 9.—JOHN WELSH, ÆT. 39. Quartan ague three months.

The above plan was tried, without effect, in this case. An aperient was given, the bowels having been confined, and the medicine then tried; but no benefit accruing, quinin. gr. v. was directed to be taken every six hours, and, under this plan, he recovered in about a fortnight.

*Case* 10.—ELIZABETH MARLE, ÆT. 40. Quotidian ague three months.

This patient was a most miserable object, the paroxysms were *very severe*; and this case, more decidedly than any of the preceding, proves the efficacy of this mode of practice.

*March* 1*st.*

*Ordered.*—R. Sulph. quinin. gr. x.

Took it about 15 minutes before the expected paroxysm: it came on at *the usual time, but was very slight.*

*March* 2*d.* Pergat—not the slightest chill.

3*d.* Pergat, no return of the paroxysm.

4*th.* The chills have not returned. To continue the medicine for a week.

I could add many more cases, but we have here satis superque.

Thus it will be seen that, however effectual Dr. Brown's plan may be, still the one adopted in these cases must be regarded as equally so: and I may state, that not the slightest unpleasant symptom occurred in any of the cases from the unusual dose. There is one advantage attached to this mode of giving the bark over that adopted by Dr. Brown, which, to those practising in districts where ague is commonly met with, will be of some consequence. It is well known that intermittent fever more frequently occurs in the poorly fed and clothed, consequently, among the lowest and most illiterate portions of the community; and I conceive it would be often so difficult to make them understand the directions and precautions necessary, if Dr. B.'s mode is adopted, that our intentions would be frustrated; whereas, in the plan here adopted, the most illiterate would comprehend, and the least persevering could not but fulfil, the one simple and only necessary direction—" to take the dose immediately before the expected attack."

IV.

TWO CASES OF STRANGULATED HERNIA, TREATED BY F. TYRRELL, ESQ. AT ST. THOMAS'S HOSPITAL.

The following cases of hernia, treated by Mr. Tyrrell at St. Thomas's Hospital, present some points of interest.

*Case of Strangulated Inguinal Hernia, in which there was some difficulty in detecting its nature.*

*Dec. 5th,* 1826.—William Poole, æt. 20, was admitted with an inflamed tumour in the right groin and scrotum. His bowels had not been relieved (according to his statement, which, however, was not very clear) since the morning of the 1st instant; and on the following day he was seized with pain in the tumour, which was quickly succeeded by nausea, with some vomiting. It appeared, from what could be collected, that he had been the subject of hernia several years. He had never employed means for its relief, and had frequently had similar attacks to the present, which had always been relieved by brandy. There was, however, one part of his statement which was contradictory—he said the tumour had never been reducible. This, in conjunction with the appearance of the tumour, and some other unusual symptoms, as will be presently seen, created some doubt as to its nature. No impulse was given to the tumour on coughing—it was painful and very tender—the swelling was not uniform from the internal ring, along the inguinal canal to the scrotum, but the swelling existed almost entirely in the scrotum, there appearing to extend from it, to the internal ring, an " indurated mass," to use Mr. Tyrrell's expression, when he afterwards saw the case, with but slight swelling, and it felt much like the thickened vas deferens and spermatic cord in ordinary cases of inflammation of the testicle. He now vomited more frequently—some tenderness and pain in the abdomen, *but this was by no means urgent*—his pulse had not the peculiar thready feel of peritonitis—it was 110. The dresser placed the patient in a warm bath—and faintness being induced, he employed, without benefit, the taxis.

Mr. Tyrrell was now sent for, and having examined carefully the case, and finding the symptoms as above described, he did not think himself justified in performing the operation without a second opinion. Mr. Callaway was therefore invited to see the case, and his opinion was entirely in unison with Mr. Tyrrell's, *viz.* that the symptoms very much resembled those of inflammation of the testicle; and, on more closely examining the tumour, they were inclined to think they could perceive fluctuation at the lower part, and a puncture was carefully made through the integuments, but only a small quantity of blood escaped.

It was now resolved to try the effects of an enema, in order, if possible, to procure an evacuation from the bowels, which, if not obtained in a few hours, it was determined to operate, in order to decide positively on the nature of the tumour. Twenty leeches were applied to the abdomen, and an enema administered about 8 o'clock vesp. and repeated in about two hours—from these, however, no evacuation was procured, and at 12 p. m. Mr. Tyrrell, with whom was Mr. Callaway, again saw the patient, and determined (although the symptoms were not more urgent than at the last report), to cut down upon the tumour, and ascertain its nature, as in a doubtful case they considered it preferable to subject the patient, even unnecessarily, to the operation, not in itself dangerous, rather than leave him to certain death, if hernia did exist.

*Dec.* 6. 1 *o'clock, a. m.*—Mr. Tyrrell made an incision, in the usual manner, from the external abdominal ring to the bottom of the tumour, through the integuments and superficial fascia, thus exposing the cremaster muscle, which, after some difficulty in introducing the director, was divided on it, bringing into view the hernial sac, into which an opening was carefully made, when there escaped about half an ounce of serum. The sac was found to contain a portion of the small intestines, of a dark colour, though not gangrenous; there was also a portion of omentum in a gangrenous state—this Mr. T. removed with the knife.

There were three distinct strictures, which were divided freely in succession—one at the external ring, another in the inguinal canal, and the third at the internal ring.

In order to examine the intestine, it became necessary to separate the adhesions which existed between it and the sac; having done which, two or three gangrenous spots were found at the seat of stricture. Under these circumstances, Mr. T. considering that the efforts of nature to relieve such cases consists in forming an opening by gangrene, he determined to adopt the plan thus pointed out, and relieve nature of this additional exertion, by making an opening himself, which was accordingly done, on which a small quantity of dark coloured fluid escaped. The intestine was left in the wound, over which a poultice was applied. To relieve the irritability of the stomach, which still continued, the following mixture was ordered:—

R. Magnes. sulph. ʒiij. aq. menth. p. ℥iss. tinct. opii, gtt. v. ℳ—tertia quaque hora sumend.

These were continued till between six and seven o'clock in the morning, when the pulse having risen and become more hard, he was bled ad deliquium (℥xij.) The vomiting still continuing, he was ordered, R. calom. gr. iij. pulv. opii, gr. j. ℳ. Stat. sumend.

9 *A. M.* The vomiting was somewhat relieved by the dose, but it soon returned, and is now as urgent as immediately after the operation. The discharge is free from the wound, but the rectum is not yet evacuated.

Cont. mist.

*Merid.* Vomiting continues — pulse 100, softer than in the morning. As no evacuation has yet taken place from the rectum, ordered,

Enema—coloc. c. stat. injiciend. et repr. si opus sit.

The enema procured an evacuation in about half an hour.

8 *o'clock vesp.* The pulse is 110, rather sharper than at last report—complains of some tenderness in the abdomen—vomiting continues—free discharge from the opening in the groin—the former medicines were directed to be omitted, and the following were ordered to be substituted:

R. Mist. efferv. tinct. opii, gt. xv. stat. sumend.—et post horas duas repetr. V. S. ad ℥x. stat.

R. Calom. pulv. opii. aa gr. j. ℳ. nocte sumend.

The abstraction of the blood produced considerable effect on the pulse—it became much more weak, and the patient appeared more feeble—he still vomits, though not so frequently—the blood is rather buffy.

*Dec. 7th,* 10 *o'clock, A.M.* He appears very feeble—his pulse is 100, and small—the discharge from the groin is very free—the vomiting as yesterday—and, as the stomach still continued so irritable, he was ordered: beef tea, ℔.j. pro enemat. R. Cal. p. opii, aa. gr. ss. ℳ secunda quâque hora.—Repet. mist. efferv.

*Vesp.* The abdomen is not so tender as yesterday—the pulse, however, being more sharp, and rather increased in frequency, a few ounces of blood were taken from the arm, which relieved the pulse, but produced greater prostration of strength—weak brandy and water was therefore given.

8*th.* His weakness is much increased—he is evidently gradually sinking—his pulse is more feeble—vomiting still continues, though the efforts are very feeble. Every exertion was made to support his strength, by brandy, wine, arrow-root, &c. ℔j. beef tea was also injected into the rectum—small doses of opium were also occasionally given—he continued sensible—the powers of nature gradually gave way, and at half past ten, p. m. he expired.

*Sectio Cadaveris.* The intestine was found free from stricture, and was of a dark colour. It was found that the lower portion of the ileum was protruded through the inguinal canal—a small portion above and below the strangulated part was slightly inflamed—the peritoneal covering was otherwise pale, with no effusion into the cavity. The mucous membrane was found considerably inflamed. Mr. Tyrrell gave a clinical lecture on the 11th December, in which he pointed out the peculiar symptoms of the case—as the unusual feel of the tumour—the peculiar state of the pulse, which, it will be observed, was more frequent, but not hard and sharp, so as to indicate the presence of peritonitis—the bowels were by no means tender or tense—nor was there the anxiety of countenance usually attendant on that inflammation.

Although the intestine was found in a gangrenous state, no hiccough was present, although we are told this is a prominent symptom.

We found vomiting, it is true, but this is not an unusual symptom in common inflammation of the testicle, which, it has before been observed, the present case, in some important points, strongly resembled.

I have before given Mr. Tyrrell's reason for making an opening into the intestine, by which he conceives (although there is some difference of opinion on this point), we assist nature materially—we do that which would eventually be effected, and thus save time.

The beef tea was injected up the rectum, from the consideration that strangulation might be high up in the ileum, and consequently not a sufficiently large surface left above for the absorption of the nutriment that was occasionally retained on the stomach. More reliance is placed on this practice in France and Germany, than in this country. It will be seen, however, from the case, that, notwithstanding every exertion, the constitution never, after the sinking first commenced, shewed any attempt to rally; and that, although the vomiting was latterly not so frequent, it appeared to arise simply from a want of power.

There is, however, one point of considerable interest, and on which there exists much difference of opinion, viz. the propriety of giving the sulph. magnes. with aquæ menth. p. in such cases as the one under consideration, to allay the irritability of the stomach, by procuring, if possible, evacuation from the bowels. It will be seen, from the detail of this case, and also from the next, that such is the practice of Mr. Tyrrell—such also is the plan of treatment adopted by some few other surgeons. But this mode of practice is by no means generally adopted, even by the surgeons of the Borough hospitals, some of whom follow the precepts and practice of the Clines, viz. leaving the patient without medicine for 10 or 12 hours, when the irritation of the stomach we are told ceases, and the cases frequently terminate favourably.

Experience only can determine this point; and while advocates are to be found for both modes of practice, it is but just to conclude, that both are, in their hands, frequently successful; but *which* demands the preference, must be decided by faithfully recording the cases, so as to afford us, eventually, just data, on which can be founded a correct opinion.

*Case of Strangulated Femoral Hernia, with the Operation and Post Mortem Examination. Treated by* F. Tyrrell, Esq. *St. Thomas's Hospital.*

Elizabeth Goodacre, æt. 64, was admitted into Ann's Ward, February 6th, about 1 o'clock, A.M.

A small tumor, to which an impulse was given on coughing, was perceptible in the right groin, immediately below Poupart's ligament. She had been the subject of hernia about 13 months, for which she had not worn a truss—a sort of bandage had, however, been applied to it.

Had not been able to return the tumor since the 1st instant; nausea and pain came on, and has continued since the morning of the 4th. She had now great anxiety of countenance, with pain and tension of the abdomen—nausea—but not actual vomiting—pulse 100, small and wiry.

The dresser tried the taxis ineffectually and then applied cold until Mr. Tyrrell's arrival, when Mr. T. again employed for some time, but without success, the taxis. There was now (9 A.M.) occasional vomiting, with continual nausea; but as the symptoms were not urgent it was determined to try an enema, and wait two or three hours before proposing the operation; accordingly, one composed of decoct. hord. c. ol. ricin. was injected, but was not retained an instant—it brought away no feculent matter, therefore, at 12 o'clock (merid.) Mr. Tyrrel performed the necessary operation to relieve the strangulated intestine.

*Operation.* Having divided the integuments over the tumor in the form of an inverted ⊥ and dissected back the flaps, several enlarged inguinal glands were found over it, firmly adherent to the fascia propria. (The hernial tumor extended more externally and not so much upwards.) Having carefully dissected to the fascia propria, this was found so firmly adherent to the hernial sac, that both were opened by the same incision.

The sac was found to contain a small fold of the small intestines of a dark chocolate color; on the outer side of the sac, there was found a small cyst which gave way under the pressure of the director, and discharged about ʒss of pus. After the stricture had been divided (which existed at the neck of the sac) in a direction upwards and inwards, or towards the umbilicus, and the portion of strangulated intestine returned—the processus vermiformis was found passing to the outer side of the sac, and connected with the cyst before-mentioned, through which, on the slightest pressure, a small quantity of apparently fæcal matter passed; this induced Mr. T. to ascertain if any communication existed between this sac and the intestines, but none could be found. The edges of the wound were brought together by a suture, and retained by adhesive straps.

*Vesp.* Vomiting continues—pain in the lower part of the abdomen increased on pressure—considerable anxiety of countenance—pulse 105, small and hard —bowels not yet open.

Venæsectio ad ℥xiv. Fotus abdomini.

R. Magnes. sulph. ʒij. tinc. opii, gtt. v. aq. menth. p. ℥iss. ℳ 3tia. quaq. hora sumend. Rep. enem. stat et rep. si opus sit.

The patient was considerably relieved by the bleeding—the pulse became softer —pain less on pressure—skin moist—not so great an expression of pain in her countenance.

*Feb. 7th.* Is much the same as at last report—little expression of anxiety or pain in the countenance—abdomen is still however tender on firm pressure—bowels not open.

*Ordered.* R. Ext. coloc. c. calom. gr. x. stat. et rep. si opus sit.

*Vesp.* The pills have been twice repeated, but the bowels have not been evacuated. Enema colocynth. stat. injiciend.

*Feb. 8th.—Mane.* Bowels not yet relieved—more anxiety of the countenance, with some increase of pain and tenderness in the abdomen—pulse 100, small and rather hard.

Rept. enema, stat. injiciend.

*Merid.* Bowels not yet relieved—expression of pain in the countenance more evident.

Repet. enema; this produced two motions, but not very copious.

*Vesp.* The bowels more painful, and much distended—pulse 100, rather feeble —more anxiety of the countenance— great thirst—skin hot and dry.

Rep. mist. fet. appl. hirud. xx. abdomini—repr. enem. cras. mane.

*Feb. 9th.* Every symptom is aggravated —vomiting has returned with redoubled violence, with increased debility. Pulse 130, very feeble—countenance appears sunk—bowels not opened by enema— some pain continues, on pressure, in the abdomen.

Rep. hirud. xij. et postea fotus abdominis, R. mist. efferves. c tinc. opii, gtt. v. sing. dos. 2da vel 3tia. quaque hora sumend. This was not retained on the stomach—vomiting increased—respiration became hurried and laborious— bowels enormously distended—patient gradually became more feeble and expired in the evening.

*Sect. Cadav.* The intestines were much distended with flatus—peritoneal coat of the intestines adherent to each other, and to that lining the parietes— the appearance of inflammation more evident as we approached the portion of ileum which had been strangulated—this part was doubled upon itself at an acute angle, and fixed by adhesions; it exhibited marks of intense inflammation. The mucous membrane of the whole alimentary canal exhibited more or less the effects of inflammation, more evident, however, in the lower portion. The appendix cæci was found firmly adherent to the cyst on the outer part of the sac, but it was quite impervious.

---

### V.

### HOOPING-COUGH.

THE two following cases were not treated in the hospital. I introduce the first, in order to add another to the list of post-mortem appearances in hooping-cough, where the brain was the only structure in the body proved to have been diseased, and shewing the necessity of more frequently directing our attention to it, as was long since insisted on by Dr. Webster. The other case (if a post mortem inspection should hereafter prove—that, of which several surgeons of eminence, who have seen it, have not, from present appearances, the slightest doubt :—viz. the existence of a lateral dislocation in the cervical vertebræ) is of considerable interest. The patient, I hope, will shortly be admitted into one of the Borough Hospitals, so that we shall have an opportunity of watching and reporting the progress of her malady.

*Case* 1. A child, æt. 4 months, previously healthy, was attacked with hooping cough, which continued for a fortnight, not very violent, for which a blister was applied to the chest by a neighbouring practitioner, to whom it was taken, and some aperient powders, to relieve the confined state of the bowels. At the termination, however, of this period, the child was attacked with convulsions, which *invariably*, from this time, *preceded* and *ushered in* each attack of the cough, and which now became more urgent; this continued, regardless of the aperients freely administered, for about three weeks, when the patient expired. I was requested to examine the body, which was done 36 hours after death.

I first carefully examined the larynx, trachea, bronchia, and lungs; but not the slightest deviation from healthy structure could be perceived. The pleura covering the lungs, and that lining the parietes of the thorax and covering the diaphragm, was perfectly healthy. The stomach and intestines, with the remainder of the chylopoietic viscera, were then carefully examined, but all possessed their natural appearance. The mucous membrane of the stomach was more particularly the object of my attention, as a physician, for whose opinion I entertain the greatest respect, believes this to be the seat of the disease, having come to this conclusion from cases which he has dissected, and in which the villi were turgid with blood, the lungs and air-passages being healthy; *but he did not examine the brain:* in this case, however, no such appearance was present. On removing the calvarium, the vessels of the dura mater were found turgid with blood, on removing which, it was found that the pia mater was also highly injected, with effusion of serum between the membranes. A great many bloody points were found on cutting into the brain, and at least two ounces of bloody serum in the ventricles.

*Case* 2. H. C. about three months since fell from the top of a stage coach on the side of her head, which "stunned" her for a time, and, on recovering her senses, it was found that the head was immoveably fixed to the left side, with considerable pain in the neck, and, on examining the spinous processes of the cervical vertebræ, that of the (?) 4th was very distinctly felt to the left of the one below it; forming not an inconsiderable angle, evidently shewing, in conjunction with the fixed state of the head towards the left side, that lateral partial dislocation, most probably conjoined with fracture, had taken place. The pain in the neck entirely subsided in a few hours, but the head still remained fixed in the same position, which it still retains. There was no paralysis or diminished sensation in the extremities for two months—her respiration natural, as were also the secretions. About three weeks since, she first felt a slight coldness and numbness in the left side, without any increase of pain in the neck, or other symptoms—this numbness continued for a few days, but did not prevent her taking her usual exercise; when, in about a week, she was attacked with spasmodic twitchings of the muscles of both the upper and lower extremities. She now applied to an eminent physician in the Borough, who found, in addition to the above symptoms, that the mouth was slightly drawn to the left side—there was inability to raise the left arm far from the side—some pain inthe shoulder and left side of the chest—pulse natural—spasmodic twitchings every five or ten minutes—general health good.

*Feb.* 17*th.* Twenty leeches were applied to the neck—pil. coloc. c. si opus sit.

22*d.* Symptoms much the same.

Cont. pil. Empl. belladon. ad nuch.

28*th.* Patient not able to attend—is reported to be much the same, as regards the twitchings—has some cough.

*March* 3*d.* Twitchings worse—cough more severe, with some difficulty of breathing—some pain in the head.

Rept. empl. bellad. Cont. pil. Mist. emulg. pro re nata.

*March* 7*th.* Cough relieved, but there is more pain in the neck, twitchings of the muscles more frequent. Some numbness in the left arm and leg.

*Ordered.*—A small blister to be applied on each side of the spinous process near the seat of injury.

*March* 10*th.* More inability to raise the arm—some swelling of the glands in the neck—in other respects as last report.

*March* 14*th.* The patient was admitted into Guy's Hospital to-day, under Mr. Morgan, No. 8, Mary Ward. Symptoms as last report—ordered to be cupped on the neck to 12 ounces—an issue to be formed on each side of the apparently displaced spinous process.

R. Ferr. carbon. ℥ss. per diem sumend.

Here our account must close, but we shall give the sequel of the case in our next Number.

---

VI.

CASE OF TETANUS, FOLLOWING INJURY TO THE FINGERS, TREATED BY F. TYRRELL, ESQ. ST. THOMAS'S HOSPITAL.

1827,

*Feb.* 24*th.* Thomas Lee, æt. 45, a stone-mason, was admitted into Edward's ward, with compound fracture of three of the fingers of the left hand—two of them (the ring and little finger) comminuted; the nails were also nearly separated from two of the fingers of the right hand. The wounds bled very freely, and the surface looked tolerably even, more resembling a cut than a contused wound, although it was caused by the fall of a stone. The man was, at the time of his admission, somewhat intoxicated; but his constitution did not, as far as could be judged from his apparent muscular strength, seem to have suffered much from drinking. Mr. Tyrrell, therefore, determined to endeavour to save the fingers. Accordingly, the wounds were carefully cleansed—several small spiculæ of bone were removed—the sores covered with lint, which was kept in its situation by slight strapping;—the fingers being, lastly, kept straight by pasteboard splints, nicely adjusted. A spirit-wash was afterwards applied, and the limb so placed, as to best favour the return of the blood. The bowels to be kept open by aperient mixture.

He suffered but little pain for several days, but (March 4th) feeling some additional uneasiness, the dressings were removed, and it was found that the ring-finger of the left hand had separated between the second and third phalanx—the first phalanx of the fourth finger was also nearly separated—suppuration was very free—the matter appeared healthy, and he again felt comparatively easy.

*March* 5*th.* Free discharge from wounds—some healthy granulations—and he continued the same till March 6th, at 7 o'clock, A.M. when he first felt a slight rigidity of the muscles of the neck, and those moving the jaw—his pulse became quickened—wounds suppurating freely.

11 *o'Clock, A.M.* Every symptom increased in violence—pulse 110, and intermitting; being, for a few beats, hard, full, and bounding,—then small and feeble—bowels not open.

R. Ext. coloc. c. gr. xx. Stat. sumend.

4 *o'Clock.* Mr. Tyrrell saw him. The rigidity of the muscles connected with the lower jaw is increased—the pulse is still very irregular—his bowels but slightly open—fæces of a dark colour.

Mr. Tyrrell enquired particularly if he had been accustomed to drink hard. He admitted taking several pots of porter daily, with an occasional dram of gin; but not, he said, to excess. He was ordered to have beef tea, ℔ij. in die. Porter, ℔j.

Repr. pil. aper. duæ. R. Calom. gr. ij. opii, gr. j. nocte maneque.

*Vesp.* Bowels freely open. Mr. Tyrrell being engaged, Mr. South has seen the patient, and, "the pulse having risen," he directed the porter not to be given. The rigidity of the muscles is now, however, much greater; and so great is the difficulty of swallowing, that but very little of the beef tea has been taken. Sometimes spasms of all the voluntary muscles are produced by the attempt at deglutition.

*March* 7*th.* Took the pills with some difficulty last evening; but the trismus is so much increased, as also is the difficulty in deglutition, that he could not take them this morning. Bowels relieved early this morning—motion dark-coloured.

Spasmodic twitchings of the muscles of the trunk and extremities now very frequent—pulse 120, irregular. At 11 o'clock A.M. tinct. opii, gtt. l. were attempted to be given by the apothecary, but without success. *Even the sight of a liquid*, or the thought of swallowing, now caused violent spasmodic twitchings.

Mr. Tyrrell saw him at 12 o'clock. The muscles of the abdomen were now rigid, and the mouth was nearly closed.

Mr. T. pressed firmly along the spinal column, but not the slightest pain was produced. He complained of no pain, except at the scrobiculus cordis ;—but, willing to grant the patient every chance, he was ordered to be cupped to 12 ozs. from the back, and afterwards blisters to be applied to the neck and between the shoulders.

4 *o'Clock, P.M.* The respiration is more hurried—pain in the scrobiculus cordis more severe—spasmodic twitchings more frequent. He gradually became worse, but his intellects remained unimpaired—and at 9 o'clock, P.M. he expired.

Mr. T. learnt from his friends, after his death, that the patient had been a very hard drinker— not having been satisfied with an "occasional dram," he had, on an average, taken a dozen per diem.

*Sect. Cad.* 40 *hours after death.*—On cutting down upon the spinous processes, (in order to examine the spinal cord) a considerable turgescence of the vessels, with, apparently, some effusion of lymph into the cellular tissue, was found under the part where the cupping-glasses had been applied. (I mention this, not having been able to learn whether this is the usual appearance presented by parts under such circumstances—if it is, what is the inference ?) The arches of the vertebræ were then removed, and a small quantity of bloody serum was found external to the dura mater ; on a puncture being made into this membrane, a considerable quantity (perhaps ℥iij. or more) of fluid, presenting the same character, escaped.

It was said, by a very minute and accurate morbid anatomist present, that the "fat found under the ligamentum subflavum was fluid, and the cellular tissue highly vascular."

The whole cord, before removing the dura mater, was of a reddish colour, presenting, particularly about the dorsal vertebræ, a number of dark blue or purple spots.

The dura mater being removed, the pia mater was found turgid with blood, and having *on it a number of small bony patches*, or *scales*, which did not adhere very firmly.

On removing the calvarium, the dura mater was found very little more than usually turgid with blood—the tunica arachnoides presented, in many parts, a milky appearance, in streaks or lines, and under this membrane a more than usual accumulation of serum. The brain did not otherwise present any thing unusual.

A gentleman present requested that the other cavities might not be opened ; it was, therefore, only possible to examine the œsophagus and larynx, which was done by removing the bodies of the vertebræ.

The œsophagus presented its usual appearance. The membrane lining the larynx was unusually vascular—there was also some œdema at the root of the epiglottis.

There appeared, on examining the fingers, to be a free exit for the matter.

*Clinical Remarks.* Mr. Tyrrell, in a clinical lecture on the above case, (on the 12th March) took a comprehensive view of tetanus, and examined the opinions of several writers on the subject, but I shall confine myself to the observations which were given as the result of his own experience, and those bearing on the case related.

Mr. T. has more frequently seen traumatic tetanus follow injury to the fibrous and nervous textures, than to any other parts—hence, its frequent occurrence after injuries to the fingers (as in the present case) or to the toes.

In reviewing the symptoms of acute tetanus, Mr. T. stated, that he usually finds the muscles of the back or abdomen first affected, after trismus—then those of the lower, and, lastly, (of the voluntary muscles) those of the upper extremity; and, *when the last become affected "death is not far distant ;"* for the involuntary muscles, and those of a mixed character, as the diaphragm and other muscles of respiration, become involved, and terminate the scene. It is worthy of attention, that the symptoms will frequently very closely resemble those of hydrophobia, as was the case in this patient. *Even the sight of water or any fluid caused violent spasms.* Taking, therefore, the word in its literal sense, the patient had "a dread of water."

Mr. T. has seen it occur at all ages, except the very old ; and in all constitutions, but more especially the young and robust. He particularly referred to the case of a delicate girl, who died at Guy's, from tetanus following injury to the fingers ; and that of a boy, equally delicate, from injury to the toes—proving that those of a robust constitution are not the only sufferers—though the most frequent.

Sometimes Mr. T. has seen considerable pain in the wound precede or accompany the tetanic symptoms; but frequently (as in the present case) no pain or uneasiness existed.

In one case, which occurred last year, pain was complained of in the cervical vertebræ, but frequently, and such was the case in the present instance, none was present; nor is there ever any cerebral disturbance.

Mr. T. now reviewed the *post mortem* appearances in these cases, in order to ascertain if these will furnish any clue to the successful treatment.

It has been lately attributed by some to inflammation of the spinal chord; and, in five or six cases which Mr. T. has examined, he has found, as in this case, effusion into the theca, and the vessels of the pia mater turgid with blood. But the spinal cord is so seldom examined, that it is impossible (with our present imperfect knowledge of its healthy appearance, coupled with the fact, that the fluids will, of course, after death, gravitate to the most dependent part) to say how far the appearance found depended on inflammation. With respect to the bony deposits which were found in this case (and which is most beautifully seen in a preparation in St. Thomas's museum, taken from a patient who died from tetanus), Mr. T. has found the same in a case of hydrophobia, and also in a case of chorea. It does not appear to him to be the product of acute inflammation, nor to have been formed in the short time the patient was affected; for, previous to a deposition of bone, it is well known that a cartilaginous deposit must take place. Mr. T. was inclined to believe, that little light has hitherto been thrown on the pathology of this disease, by post mortem examinations.

How, then, are we to treat it? Are we to bleed, as some have recommended, or should we stimulate, and give antispasmodics, as others have as strongly advised? Mr. T. thinks that each plan is adapted to particular cases, but that it is impossible to legislate for all.

The state of the wound will naturally first claim the surgeon's attention, and if there be pain, examine it attentively, as probably some spiculæ of bone or collection of pus may be keeping up an irritation. if it is found impossible, as in a severe comminuted compound fracture, to remove the spiculæ, amputate the limb. But it will, as the following case illustrates, sometimes be in our power to remove the cause of irritation, without resorting to this measure.

A patient was brought to St. Thomas's Hospital, and admitted under Mr Tyrrell, with compound dislocation of the great toe. The end of the bone protruded. This was reduced; but trismus came on, and he could not swallow without difficulty. Dr. Elliotson saw him, and ordered, fer. carb. ℥ss. 2nda. vel 3tia. quaq. hora. He continued this for eight days—the disease gradually increased—and at this time the muscles of the abdomen had become rigid. Fluctuation was now felt on the dorsum of the foot; a free incision was made, and about ℥iij. of a fetid pus escaped. The patient quickly became better, but the iron was continued in smaller doses for some time.

Some may be inclined to attribute the cure to the iron; but Mr. T. thinks it probably prevented the disease proceeding more rapidly; yet, until the matter was evacuated, the disease gradually increased.

Sometimes the discharge from the wound is altered, or stopped altogether; in this case, Mr. T. recommends counter-irritation in a neighbouring part, which will, in a few hours, restore it. This is founded on experience. He finds *blistering* the best mode of counter-irritation.

The bowels will frequently be constipated, as was the case in this patient; these must first be relieved—the other secretions and excretions must also be attended to.

Mr. T. considers it a *most important* point in the treatment of these cases, and of injuries generally, to ascertain the previous habits of the patient. Although this man appeared stout and muscular, still, had he been aware of his previous habits, he would have allowed him a much greater quantity of stimuli. To illustrate this proposition, he related the two following cases.

A man was brought to the hospital, somewhat intoxicated, with considerable injury to the knee-joint. Mr. T. recommended amputation, but the patient would not then consent. On the following day, however, the operation was performed, and the stump was dressed in the usual manner. He became delirious, and his pulse was irregular. Mr. T. now learnt that he was accustomed to drink largely. Some porter was immediately sent for, and, although delirious, the man was permitted to drink freely. He went to sleep—his pulse became calm and regular—and he recovered.

Another patient, from injury to the elbow, had diffuse cellular inflammation. He became delirious. It was found, as in the former case, that he was accustomed to drink freely. He was allowed porter—the delirium subsided—and he recovered.

Thus, in short, Mr. T. recommends the surgeon to be guided by the constitution and state of the patient. If robust and young, bleed freely, open the bowels, and afterwards give opium, or perhaps camphor and musk. If he has been accustomed to stimuli, give that to which he has been most accustomed, combined with antispasmodics—attending, in all cases, to the secretions and excretions.

---

### VII.

### OPERATIONS.

*Amputations above the Knee, by* B. TRAVERS, Esq.

JOHN LINGARD, æt. 20, was admitted under Mr. Travers with chronic inflammation of the knee-joint.

The patient's strength being supported by nourishing food, with decoct. cinchon. c. acid sulph. leeches were repeatedly applied, with fomentations and poultices, to the knee. After this, counter-irritation, by the ung. ant. tart. was tried, which relieved the pain, but the swelling continued. An attempt was made to relieve this by pressure, which is said sometimes to have succeeded. Rollers and splints were applied; but without affording any relief. The swelling, indeed, increased —the patient's constitution began materially to suffer—and, on March 2nd, the limb was removed by the circular incision.

On examining the joint, after its removal, the surrounding integuments, &c. were found indurated and thickened. On opening the joint, a considerable quantity of thin pus, mixed with a curdy matter, escaped. The semilunar cartilages were destroyed by ulceration, as was also the ligamentous structure covering the condyles of the femur, and the articulating surfaces of the patella. The extremities of the femur and tibia were not enlarged; but were of a softer texture than natural. The synovial capsule was thickened.

GEORGE POCOCK, æt. 35, was admitted (August 24th, 1826) into Isaac's ward, under Mr. Travers, with chronic inflammation of the knee, which had existed several years, and was first induced by a fall, for which he had been under the care of various practitioners, during nearly the whole period since the receipt of the injury.

The same plan of treatment, with the exception of pressure, was employed in this, as in the former case, with apparently some benefit. But, after submitting to a confinement of more than six months, the patient became extremely anxious for the removal of the limb; feeling, he said, but little hope of ever again being able to use it; as, even now, the slightest pressure on the foot caused considerable pain, although none was felt in the recumbent posture. At length, Mr. Travers, with the concurrence of Mr. Green, consented to remove it, which he did in the same manner, and on the same day, as the case above.

It was found necessary, in this case, to take up the femoral vein.

*March 15th.* The patient is going on well.

On examining this joint, it was found to exhibit a beautiful specimen of incipient anchylosis. The crucial ligaments were removed by ulceration and absorption, as was also the central part of the semilunar cartilages, so as to form a deeper cavity to receive the articulating surfaces of the femur, the form of which had become so modified, as to be more accurately adapted to the tibia. A callus had been thrown out, uniting the extremities of these bones, in which ossification had commenced at several points, on the posterior part, where there was also one spot of ulceration remaining. The patella had become similarly attached to the femur.

OPERATIONS, BY J. W. GREEN, ESQ.

*Amputation at the Shoulder Joint.*

Elijah Bird, æt. 13, was admitted under Mr. Green, Sept. 6th, 1826, with extensive ulceration of the left arm, and anchylosis of the elbow joint.

It appears that, more than 2 years since, his arm was severely burnt by his clothes taking fire, when he was taken to St. George's Hospital, where he remained some time without receiving any benefit.* He was then removed by his friends to St. Pancras Infirmary, where he remained several months. On his admission into the infirmary, he had anchylosis of the elbow joint, and the ulcer was about the same size as on his admission to St. Thomas's; when it extended, on the anterior part, from above the insertion cf the deltoid, nearly to the wrist. Behind, it did not extend so high; and, near the elbow posteriorly, the skin was entire.

It would require much more space than is allotted to me, to decribe the various applications that were tried. Suffice it to say, that nothing produced even a tendency to heal, if we, perhaps, except the last application; which was the formation of a thick eschar by means of the lapis calaminaris. This was perseveringly employed for some time, and the ulcer showed some disposition to heal round the edges; but this improvement was very slight, and the patient became so extremely weak, from the continued discharge, with an inability to take sufficient nourishment, that nature must inevitably have soon failed under the exertion. It was therefore, as a last resource, determined to remove the arm at the shoulder joint, it being impossible to save sufficient integument any lower in the extremity. Accordingly, Feb. 16th, Mr. Green performed the operation.

The patient was seated in a chair, and on examining the limb, it was found that not sufficient integument could be saved for a flap anteriorly. It, therefore, became necessary to save it from the sides and behind.

Mr. Travers compressed the artery very effectually below the clavicle, it being found impossible to do so above, as the patient was unable to bear the pressure.

Mr. Green then made an incision, commencing from just below the insertion of the pectoralis major and to its inner side, extending it to the acromion process of the scapula; then proceeded from this point downwards and backwards, to the outer margin of the axilla (thus leaving attached to the arm a triangular portion of the coverings, the apex of which, was at the coracoid process) the flaps were dissected off to either side—the anterior part of the capsular ligament was divided, and the elbow depressed, in order to throw the head of the bone from the glenoid cavity; but this was impracticable, from the soft anchylosis that had taken place. A catlin was therefore passed under the head of the humerus, and brought out between it and the glenoid cavity; then, by one sweep of the same instrument, (which was again introduced behind the head of the humerus) the muscles on the back part, to as low down as the base of the triangle, with the vessels and nerves, were divided, which completed the operation. The vessels being secured, the flaps on each side were brought together by adhesive straps.

*March 15th.* The patient is doing well.

*Lithotomy.*

1827.

*Feb. 15th,* John Bone, æt. 65, was admitted into Isaac Ward under Mr. Green, with symptoms indicating stone in the bladder, which, on sounding, was found to be the case.

*Feb. 23rd.* Mr. Green having again sounded, performed the lateral operation. The transversalis perinei artery bled rather more freely than usual, and there was also a more than ordinary gush of venous blood; but not sufficient to prevent Mr. G. proceeding steadily in the operation, and extracting the calculus; having done which, he secured the artery by ligature.

The instrument used was the gorget,

* We recollect seeing this lad at St. George's, and we can state that a great deal of attention was paid him, and a variety of applications tried without success. At one time, under the use of the sulphate of copper (we believe) cicatrization took place to some extent; but the boy was of a miserably scrofulous habit, and the sore so extensive, that there was not the least chance of its ever permanently healing.—*Ed.*

which, in the hands of Mr. Green, has, in *upwards of* 70 *cases, proved invariably successful.*

The patient gradually recovered. The urine now (March 8th) passes entirely by the urethra; and he is allowed to sit up a short time.

A circumstance occurred, about a week after the operation, which affords a useful lesson. The patient's bowels had been allowed to become rather costive, and he passed some hardened fæces, with some difficulty. At this time, the urine nearly all passed by the urethra; but this exertion separated the slight adhesions, and it again passed freely by the wound, which, of course, somewhat retarded his recovery.

### Operations, by F. Tyrrell, Esq.

#### *Popliteal Aneurism.*

1827.

*Feb.* 1*st*. George Graham, æt. 32, of spare habit and debilitated constitution, was admitted under Mr. Tyrrell, with very distinctly marked popliteal aneurism. On further examination, in order to ascertain if any other existed (which Mr. T. invariably does before proposing the operation, and the plan is worthy of imitation) he thought, as also did Mr. Green, that he could distinctly feel an aneurism at the bifurcation of the aorta. The operation was therefore not proposed, till, on examining very attentively a few days after, no traces of the supposed aneurism at the bifurcation could be felt. This examination was again repeated, a day or two afterwards, with the same result. Mr. T. now ascertained that he had an anterior curvature of the spine, and thought it probable, though by no means certain, that the aorta being thrown forward by the curvature, and the patient being, on the first examination, agitated, it was the vessel itself which was felt pulsating.*

*Feb.* 6*th*. The femoral artery was taken up in the usual place (where it is crossed by the sartorius muscle) and it completely commanded the pulsation in the tumour, which has gradually diminished, and is now, (*March* 10*th)* not perceptible. There is still, however, a numbness in the extremity; but the temperature of the limb is natural.

The ligature has separated, and the wound in the thigh is nearly healed.

#### *Amputation.*

Dennis Burns, æt. 29, was admitted, under Mr. Tyrrell, into William's ward, July 29th, complaining of a long-continued pain at the upper and anterior part of the head of the tibia, where, on examination, an elastic tumour was perceived, on pressing which the pain was not increased.

Strict antiphlogistic measures were instantly adopted. By bleeding locally and generally, frequently repeated, followed by counter-irritation, the pain was nearly conquered; but the swelling still continued, and, at length, (in about five months) the integuments covering the part ulcerated, and a considerable quantity of bloody matter escaped. On examination, it was found that the ligamentum patellæ was ulcerating, and the tubercle of the tibia partially exposed. The ulceration extending, and the patient's strength suffering materially, (although every exertion was made to support it, by nourishing food and tonics) it became necessary to remove the limb, which was done, by Mr. T. on the 9th of March.

There was nothing worth remarking in the operation, if we except, perhaps, the necessity of taking up the femoral vein.

*March* 15*th*. No pain in the stump—patient is going on well.

*Examination of the Limb.* The ligamentum patellæ was found completely ulcerated through, so that the rectus, crureus, and vasti muscles, having nothing to antagonize them, had drawn the patella above the articulating surfaces on the femur. The tubercle of the tibia was exfoliated to a considerable extent. Although so much mischief had been produced in the immediate vicinity, the joint was perfectly healthy.

THOMAS H. SMITH.

*London,* 15*th March,* 1827.

* After repeated examinations, however, since the operation, the tumour has at length been again felt at the bifurcation, and on the application of the stethoscope, the peculiar "thrilling" or "whizzing" sound, denoting an aneurism, can now be distinctly heard. T.H.S.

N. B. This whizzing sound is now acknowledged to be no positive proof of aneurism.—*Ed.*

II.

*To the Editor of the Medico-Chirurgical Review.*

Sir.—Dr. David Barry has published, in your Number for January, (p. 292) an appeal to the "literary integrity of the country," and a demand of an "*amende honorable*," by which, it may be presumed, he understands an apology, from the editor, or the author of a criticism on his "Researches on the Influence of Atmospherical Pressure," published in the Edinburgh Journal of Medical Science for October last.

The following seem to constitute the whole grounds stated by him for this not uncommon claim on the part of authors who feel themselves ill at ease under the remarks of their reviewers. Dr. B. complains—

1, That the writer alluded to has not employed Dr. Barry's own words in delivering a brief view of his doctrine.

2, That the six propositions in which he comprehends that condensed view are not *pure : i.e.* are mixed up with foreign matter.

3, That the reviewer "charges his book with asserting, that atmospheric pressure is the sole cause of the blood of the veins moving towards the heart, and has also charged the illustrious philosophers, Cuvier, Dumeril, &c. with abetting this assertion."

4, That the reviewer has committed the latter misreport, by mistaking the application of the French word "*uniquement*."

5, That the reviewer has misapplied a calculation which he amplifies from Haller, with respect to Dr. Barry's theory.

6, That the reviewer has given a physiological misrepresentation, and (if the expression be intelligible) has written disrespectfully of the great names of Cuvier, Dumeril, and Laennec.

These are grave charges, six of which, had we by any chance become guilty, we should not, for a moment, have hesitated to undergo the "amende honorable" called for by Dr. Barry, even in the strictness of the letter. They implicate our character as judges, as fair and honourable men: and, necessarily, suggest what Dr. Barry has presumed, that we have neither sense to understand his book, nor honesty to render a fair account of it to that tribunal which we are not less anxious to please than he is himself. We shall endeavour to shew him, in a few words, however, that the accusations are entirely void of foundation; and, in doing so, we hope he will excuse us if we pass over a number of lesser charges made by him, in the same *reclamation,* and which affect neither our industry nor our good faith, without reply or retaliation. Thus, it is of no consequence to the public whether the reviewer be "nameless" or renowned, provided he have spoken the truth. Neither does it matter much that he hath written, after the manner of other such critics, in the plural number: nor that he thought proper not to perplex a simple numerical calculation, "by applying to it the lights of modern science:" still less would your readers be edified by learning, that the Edinburgh reviewer most heretically believes in the infallibility of the multiplication table, applied as above, and that he aggravated this offence by writing Q. E. D. at the end of this arithmetical argument. We must explain, however, to the unmathematical part of your readers, that Q. E. D. are not the initials of *Quod Extingerit Davidem,* as some have supposed, but a common contraction the reviewer learned at school, for *quod erat demonstrandum.* In fine, your readers will care nothing for being told the reasons why the reviewer speaks of "sugescent power, suction, rotundity," &c. and occasionally in a very elliptical manner, as a man may sometimes be supposed to do, who is forced to condense the marrow of a book of 175 pages, and his own criticisms, into something less than three pages of a periodical.

We proceed, then, to the major charges-

*Charge* 1. The reviewer did not employ Dr. Barry's phraseology, for two reasons. He preferred his own; and he could not have condensed that of Dr. B. into less than a sheet and a half.

*Charge* 2. The six propositions, with their authorities now added, are as follow.

1. The moving force of the arterial blood, whatever it is, becomes extinct in the extreme arteries.

Authorities.—"As the blood passes through the greater veins during inspiration only, while it is incessantly traversing the arteries, it follows that an accumulation must take place somewhere between these two orders of vessels." 36.

"There are three quantities of blood: one passing through the arteries, one which is sucked up by each expansion of the thorax, and a third which is collected between these two points." 36.

2. The movement still exhibited by the blood in the veins, is entirely owing to the sugescent power exerted by the right side of the heart.

Authority.—*See the answers to the second charge.*

3. This sugescent power originates in the removal of atmospheric pressure by inspiration.

Authority.—*See the same as in the last.*

4. We need not repeat this and the following three propositions, which refer merely to the influence of diminished atmospheric pressure upon the absorbent system, according to the views of Dr. Barry, and are so evidently paraphrases of his notions, that we must take the liberty of requesting him to favour them with another perusal. He will then see good reason to praise, rather than to impugn their comprehensive accuracy. As Dr. B. has only stated the *impurity* in one of these six propositions, namely, the second, it may be inferred that the impurities of the other five are merely illustrative, which afforded him no argument against his reviewer, and, therefore, however much they may offend the purity of the Doctor's taste, call for no reply.

The second proposition is asserted to be vicious in attributing to Dr. Barry the doctrine, that the power by which the blood in the veins is moved onward to the heart, *is solely* the result of atmospheric pressure. That gentleman has not deemed it necessary to state, in his reclamation, *how far* this was from his real opinion when he wrote the Researches, and from the tenor of the researches themselves. We shall, therefore, lay a few short sentences of that work before your readers. "Of these supposed powers, (*sources of impulse to the blood*) some are so little susceptible of being demonstrated by direct experiment, others must be so uncertain in their operation, and the theories which they have been brought to support are so opposed to each other, that the evidence against is, *à priori*, nearly as strong as for their existence." 3.

Atmospheric pressure is not a "secondary agent," p. 5. "The blood which runs contrary to its own gravity arrives at the heart only during inspiration; and the power which impels it at this moment is atmospheric pressure," p. 35. But circulation goes on in any position of an animal—nay, the heart of an animal may easily be made the highest point: consequently, the atmospheric pressure becomes, in such a case, the sole moving power of the venous blood!

Again, "Of these powers (which move the blood), the pressure of the atmosphere is by far the most intense in its degree, the most constant in its influence, and the most unvarying in its amount. It is that, without which the circulation could not be maintained beyond a few moments." p 57.

Where then, and we appeal to Dr. Barry himself, are we to find an expression for the force of those *other* powers? The arguments against their existence are as strong as those in their favour; they could only act for "a few moments" if they act at all; and they might be annihilated, by making the heart the highest point of the system, and are always annihilated when opposed to gravity. They are, therefore, less than that force, which, however, in its most efficient mode of influence, produces NO sensible effect upon the functions of circulation, and are, therefore, less than any assignable quantity. Where, then, was the impropriety of considering them as nothing, in a general view of the theory of Dr. Barry? They are efficiently nothing in Dr. Barry's own words; and that every one else understood the Doctor in the same sense, the following passage, from Dr. B.'s own translation of the report to the Institute, as published in his Researches, will sufficiently demonstrate. To make verbal quibbles upon the difference between a *sole* cause and a *principal* cause, coupled with other smaller causes, of which the effect is insignificant, would be *nodum in scirpo quærere*—in short, to make a distinction without a difference.

Atmospheric pressure is not merely "an auxiliary mean of facilitating the arrival of the venous blood," p. 65 & 16. Dr. Barry has "fully ascertained that the sucking action of the great veins was precisely coincident with the instant when the animal endeavoured to form the vacuum in his chest; that THE BLACK BLOOD PASSED THROUGH THE VEINS ONLY DUR-

ING THE ACT AND TIME OF INSPIRATION: and that this venous movement was *always* placed under the influence of the action of atmospheric pressure." p. 69. "Upon a comparison of the relative velocities of venous and arterial blood," the author founds the notion, that "the pressure of the atmosphere is the *principal power* which impels the venous blood to the heart during inspiration." p. 70.

We must declare, that the mere idea of the pressure of the atmosphere being the principal cause, was not first taken up by him." 71.

M. Barry attributes the dilatation of the heart itself, and of its auricles, to the tendency to a vacuum, which takes place in all the cavities of the chest during inspiration," p. 71.

The report from which these extracts are taken, is signed by Cuvier and Dumeril, and, with the approbation of the Academy, countersigned by their secretary, Baron Cuvier. The Edinburgh critic was not, therefore, alone in this only rational interpretation of Dr. Barry's memoir.

*Charge* 4.—Is but a grammatical one—a conjecture that the reviewer of Dr. Barry mistranslated the French word "uniquement," in Dumeril's report. That word occurs only thrice in the report; and in the two which Dr. B. has thought proper to translate, he expresses its sense in English, by the word *solely.* In that sense, precisely, was the word understood by the reviewer; and he also thought he understood why Dr. Barry, in his translation, chose to *omit it* in the passage below. "In fine, although the greatest number of physiologists attributed the progression of the venous blood towards the heart (solely), to a vacuum formed in this organ," &c. p. 65.

The omission is such as a man would make, who was very anxious to secure the patent right of this sole propelling power to himself, and who trusted to the good sense of his readers for their avoiding the *bore* of comparing his translation with the original! At all events, his reviewer has no where made the least use of the word "uniquement," either for or against his theory, and therefore it can be made no fair ground of complaint by the author.

*Charge* 5. The reviewer has misunderstood and misapplied the calculation of Haller.—Let us see. Dr. B. himself confesses, that, by approximation, it shews how little the pressure of the air can impede the current in the pulmonary arteries! It therefore must afford us approximate measures of the force of that pressure, and of the momentum of the blood rushing through the lungs, and, consequently, of their proportion to each other. The calculation, to be sure, only affords a *mininum* proportion, and is distinctly stated by the reviewer to express merely, that the relation of the diminished atmospheric pressure to the action of the right ventricle is not greater than 1 to 355. The ratio ought to be increased a little, by the difference between the velocity of the blood in the vena cava, and in the pulmonary artery, but the result would only produce a trifling difference, not worth the pains of multiplication. But had the reviewer been even as much in the wrong as he is in the right, Dr. Barry had no more reason to demand satisfaction for his belief in the doctrines of Haller, than for his being a presbyterian.

*Charge* 6. Misrepresentation and disrespect of Cuvier, Dumeril, and Laennec. The only sentence in which they are alluded to, except barely for the purpose of being named, is this: "It would seem, even the illustrious Cuvier, Dumeril, and Laennec, still persist in attributing the *whole* of an effect to respiration, of which it can but at most, in the nature of things, produce the $\frac{1}{355}$ part."—Edin. Journ. Med. Science, No. V. p. 463. The passages formerly extracted from their report will enable the reader to perceive, that there is virtually no overstatement here: and he will possess extraordinary tact, if he can pervert any portion of it into incivility or disrespect. To these eminent foreigners we owe merely the tribute due to genius and science, respect, and we pay it cheerfully, but we see not how it should ever prevent us from stating the simple fact.

We have now refuted the whole of Dr. Barry's charges with little trouble. We wish, indeed, he had rather entered on the argumentative part of the review he condemns, than amused himself and us with groundless imputations, and clamour for an "amende honorable," which he must now see is due by him to us for his unsound doctrines and unseasonable interpellation. His vacuum drum had long sounded, because it was empty—but now that it is riddled and tattered, let him stand by it like a good soldier, and we shall give him fair battle on any point of our argument he may choose to impugn as the weakest. We never counted any thing on the Hallerian computation, farther than as a shrewd guess at the greatest effect of the atmospheric pressure—and we did not even number it among the other four heads of arguments which we consider as irrefragable proofs of the insignificance of the latter. Dr. Barry, equally, by his silence, seems to consider them as unanswerable. Should he change his mind, however, he will find us quite ready to discuss the question with him in a proper manner, for we love physiological investigation, when the object of the controversy is neither gain, nor the glory of victory, but a genuine search after truth. In short, we say that atmospheric pressure is almost nullity, and sheer nonsense.—Let the Doctor disprove our position. We are, &c.

The Reviewer of Dr. Barry, in the 5th Number of the Edinburgh Journal of Medical Science.

---

III.

*Case of Dropsy of the Pericardium, in which the Operation of Tapping was performed.* By Mr. Jowett.*

*Nottingham, March, 7th,* 1827.

My Dear Sir.—You have probably learnt, from the newspaper paragraph, or the Lancet of last week, into which it is copied, that I have ventured to perform the operation of puncturing the pericardium, in a case of effusion arising from pericarditis. Under the impression that a sketch of the case would form an acceptable communication for your valuable Journal, I forward the following particulars. It may first be necessary for me to premise, that, in consequence of several examples of latent pericarditis, as well as several instances of adhesion of the pericardium to the heart, having fallen under my observation in the early part of my pathological researches, my attention had been particularly directed to the detection of that insidious and not unfrequent disease. When Collin's excellent little work, "Des diverses Methodes d'Exploration de la Poitrine," appeared, I hoped to have found a valuable stethoscopic indication in the "*bruit analogue, ou craquement du cuir neuf,*" which he mentions as a probable sign of the first stages of pericarditis. The year 1825, however, passed without my being able to verify that writer's statements in a single instance; and as Laennec has not even alluded to the phenomenon in the late edition of his work, it may be presumed, at first sight, that the hints which Collin threw out have not been substantiated by subsequent observations.

In the course of last winter, two fatal examples of copious effusion into the pericardium, from inflammation, fell under my observation, and, from them, I acquired some valuable data, as to the extent to which the membrane may be distended, and the indications of auscultation and percussion; and I also satisfied myself, that, where there is copious effusion into the pericardium, the bag may be punctured anteriorly without danger to the heart, as that organ, under such circumstances, is pushed upwards and backwards, towards the root of the bronchia.

Last spring, I met with several cases of acute pericarditis, denoted by general symptoms, and in which I also observed, for the first time, a peculiar *rustling kind of noise,* seeming to accompany and to be caused by the auricle's action, though not exactly synchronous with it; which these and subsequent observations lead me to regard as a *pathognomonic sign* of a layer of coagulable lymph effused upon the pericardium and heart. This phenomenon is always the most distinct in the superior part of the præcordial region, the cartilages of the third and fourth ribs being the situation where it is generally the strongest: though it appears to occur during the action of the auricles, it does not accompany it exactly, but seems rather to arise from the general action of the organ: it is irregular; varies much in degree and character at different times; and sometimes disappears altogether for intervals of various duration. It approaches the nearest to the *rushing noise* of diseased valves, but an experienced ear readily distinguishes the latter by its regular uni-

---

* In a letter to the Editor.

Lancet 11:712, 1827

form character, and by its duration according exactly with the systole of one or both of the heart's cavities, whilst the former is various and irregular—indeed, the term *rustling*, which I have employed, gives a better idea of the noise than any other word. The *noise of muscular action*, respecting which Laennec says so much in his second edition, is so obviously different, that no accurate ear can be at any loss, after having once heard both the phenomena.

Last June, a case of acute pericarditis came under my care, in which the accumulation of fluid interrupted the heart's action so materially, that I expected the issue to prove fatal, unless it were evacuated by the operation. I at that time inserted the following observations in my note book.

"*June* 14*th*, 8 *A.M.* I attribute the interruption of the heart's action to the fluid filling the pericardium; and from a careful examination by the stethoscope, percussion, &c. I think the pericardium might be cautiously punctured with a trocar between the 6th and 7th left cartilages, without wounding the heart, and the fluid be abstracted. At the same time, I consider that, in the present stage of our knowledge, the operation would only be justifiable under desperate circumstances, and, when it is clear that death must ensue without it, and I have mentioned it to the friends as a measure it might be right to try, as giving a chance of life, when no other chance remained. Should the case become desperate, I think I should do it, if he consented.

"In the performance of the operation, I think two things are essentially necessary to be guarded against. (1st.) Not to wound the heart, and (2) not to admit air into the pericardium.

"1. An instrument introduced between the cartilages into the pericardium, in the natural state, where there is no fluid, would be almost certain to wound the heart, as it is in immediate contact with it. When, however, there is considerable effusion into the pericardium, I have observed that the heart is pushed backwards, and also upwards, towards the root of the bronchia, whilst the inferior anterior part of the bag is occupied by the fluid;—consequently, a trocar might be introduced with safety.

"I consider it essentially necessary to prevent the admission of air into the pericardium for these reasons. 1st. To admit air instead of fluid, would only be to substitute one cause of obstruction to the heart's action for another. 2nd. That from facts I have collected, in cases where paracentesis thoracis was resorted to, I am satisfied that the admission of air occasions the decomposition of the fluid left in the cavity, (and it is impossible to remove it entirely) which then becomes putrid and acrid, and excites fresh inflammation; and thereby destroys the chance of success, if the case be urgent previously. I think the syringe-pump, adapted to a trocar, would effectually prevent the admission of air."

I have been thus particular in stating these circumstances, in order to shew that I had fully weighed and resolved upon the propriety of the operation, long before I had occasion to resort to it. I will only add, before I proceed to the case, that the patient whose state elicited the above remarks, began to improve the same day, and ultimately recovered by the use of other remedies.

On Jan. 12th, 1827, I first visited John Skinner's daughter, aged 14 years, who was then labouring under a severe attack of acute rheumatism for the fifth time. The general symptoms induced me to suspect that there was pericarditis likewise, and on examination with the stethoscope, the *rustling* noise was audible about the nipple—there was a dull sound on percussion in a large præcordial space—the heart's action was indistinct, and apparently distant. On the 15th, I gave my diagnosis "*pericarditis with considerable effusion*."

Under the use of general and local bleedings, blisters, antimonial and mercurial medicines, and active purgatives, much relief was obtained; but she was troubled with a violent cough, occasioned by severe acute inflammation of the bronchia, which greatly aggravated her sufferings. On the 25th, both the legs were observed to swell much when she was got out of bed, and the ankles became œdematous. On the 27th, she began to take tartarized antimony in doses of three fourths of a grain every third hour, and which she continued until she had taken eighteen grains, without any vomiting being excited. It produced a decided beneficial effect upon the bronchial inflammation. On Feb. 1st, she was "much better—slept several hours in the night, and feels much refreshed—breathing greatly relieved, and the cough is now quite easy compared with what it was. The heart's action is strong, forcible, rapid and extensive, and seems rather mixed with some roughness of sound. Dry bronchial rattles in both lungs anteriorly—vomited this morning after taking tea," (for the first time.)

From that time, for a week or more, there was a manifest improvement in every respect except the œdema of the lower extremities. Feb. 12th, I reported "very ill last night—says 'she seemed as if something was strait on her stomach and tied up her inside, and prevented her getting breath, and lying down in bed:' she feels better when she sits up—the ankles and legs are worse swelled. *Effusion must have increased in the pericardium.*" Next day (Feb. 13,) the breathing was quite as bad—"dead sound (on percussion) in a very enlarged præcordial space"—there was likewise a distinct *rushing noise* at the bottom of the left side of the præcordial region, which induced me to add to my former diagnosis, "*deposition of lymph on the mitral valve?*" proposed tapping the pericardium if she became worse.

The following day she was so much worse in all respects, that it was evident she could not long survive, unless some relief were given. The face, as well as the extremities had become œdematous—respiration was impossible in any other posture except the erect, and there was much accumulation of mucus in the trachea. The operation having been consented to, I performed it in the following manner, the same afternoon, in the presence of Dr. Manson the consulting physician, Mr. Robert Jowett, my brother and pupil, and the patient's friends. Having made a small incision with a lancet through the integuments, between the 5th and sixth cartilages, exactly half way between the sternal extremity of the 6th rib, and the middle of the ensiform cartilage, I thrust a trocar, which had its canula guarded, so that it could not penetrate further than one inch, directly through the thoracic parietes; as I withdrew the trocar, two or three drops of serum escaped, and before I could adapt the pipe of the syringe apparatus to the canula, a little air was sucked through it, during the act of inspiration. On attempting to work the syringe, no fluid was abstracted; imagining, therefore, that I had not punctured the bag, I again introduced the trocar, but with no better success. Certain of the correctness of my diagnosis, I then determined with Dr. Manson's concurrence to make another attempt higher up, where there could be no possibility of missing the pericardium, and I accordingly repeated the same process between the 4th and 5th cartilages, as near the sternum as there was space enough for the instrument to pass. Here the trocar seemed to push something before it which it did not appear to penetrate, and although, here likewise, I twice introduced the instrument, still no fluid could be sucked out by the syringe.

I left the patient under the impression that I had failed in the operation—and my prognosis was "*death in the course of the night.*" Late in the evening, I found her quite as bad, *but no worse* than before. On the following morning there was an evident amendment—the breathing was less laborious—she coughed more strongly—pulse 126, fullish—the right leg was less swelled, and she had made *two quarts* of urine in the preceding sixteen hours. In the course of that day she made 5 or 6 pints more water—the œdema of the lower extremities decreased rapidly, and the improvement was rapidly progressive in every respect except the cough.

I now perceived, from weighing all these circumstances, that the operation had really been performed, so far as regarded the penetration of the pericardium by the trocar, but in consequence of the canula having been introduced but a little way, it either had never entered the bag, or else had entered so short a distance as to slip out again immediately on the withdrawal of the trocar. Acting on this view, I renewed every exertion, and the improvement from that time, for eight succeeding days, was such as to warrant great expectations of ultimate success. Weakness was the most threatening circumstance, and that was attempted to be obviated by tonics, wine, and mild nutritious food.

On the 20th Feb. the sound on percussion was dull, only in a moderate sized præ-

cordial space, and the heart (examined by the stethoscope) had returned to its natural situation. Feb. 22, she felt better—"not so low nor so faint—rested well in the night—appetite improving—tongue clean—pulse quick—ankles a little swelled." She partook more freely of food. The next day, (Feb. 23,) being the tenth from the performance of the operation, my report ran thus "has had much pain and soreness under the right ribs since last night, and has, twice, this morning vomited green slimy fluid—bowels rather costive. *Hepatitis?*" She sunk exhausted in the course of the afternoon.

At my earnest request, seconded by that of Dr. Manson, whose kindness on this and other occasions call for my warmest acknowledgments, the friends permitted an examination of the body to be made, at which Mr. T. R. Tatham also assisted. The exact situations of the punctures were found to be as before described. The *diaphragm* was drawn very high up into the thorax, so that if a pointed instrument had then been introduced perpendicularly at the place of the lowest operation, it would have been punctured. The *pericardium* adhered externally to the anterior part of the left thoracic parietes, through the medium of a layer of firmish recent lymph; and internally it was found every where adherent to the surface of the heart and large vessels, by means of a similar layer of lymph, which varied much in quantity in different places, being in some parts from one-eighth to one-fourth of an inch thick. The adhesion was moderately firm, and the lymph of a reddish colour. As the cavity of the pericardium was destroyed by this state of things, there was of course no fluid remaining in it. The pericardium proved to have been perforated at both the points of operation, and there were two holes at each place, so that every time the trocar was introduced, it had penetrated the bag. On the surface of the right ventricle, opposite the upper or last made puncture, there were two dark spots, which, on examination, proved to be drops of coagulated blood enveloped in the layers of lymph, and which had doubtless come from the wounds of the pericardium, as the surface of the heart was untouched. Both the ventricles and auricles were of the natural size. The muscular texture of the heart was rather flabby, and paler than natural. The edge of the mitral valve, on its auricular surface, was beset with a small ridge of semi-cartilaginous lymph, evidently of recent deposition, although firm and hard.

To this long account I may add, that I have since tried the experiment of puncturing the free portion of the pericardium on the dead body, and I have found, that the point of the trocar readily penetrates the sac; but as soon as the edge of the canula (which of course is always rather larger than the instrument it contains) comes in contact with it, it pushes the pericardium before it, and does not enter it, unless it be introduced to a considerable depth. This explains the supposed failure of the operation in the first instance.

I conclude by observing, that the following propositions appear to me fair practical deductions from the case:—

1st, That the operation of tapping the pericardium may be performed without injuring the heart, or endangering the life of the patient.

2nd, That the operation affords a probable chance of saving life, when all other means have failed.

3rd, That it is proper and justifiable under urgent circumstances.

Believe me, Dear Sir,

Your most obliged servant,

THOMAS JOWETT.

---

### IV.

### *A Short Outline of the* NEW MEDICAL DOCTRINE *of Italy; in a Letter from a Medical Gentleman abroad, dated Jan. 27th,* 1827.

[Communicated by Dr. B. of B——n.]

THE doctrine of contra-stimulants had its origin in the year 1799, during the prevalence of an epidemic fever at Genoa, and at a time when Brown's doctrine of excitability had numerous followers among the medical schools of Italy. Such ill success had those who followed Brownonianism in the treatment of fever, and so opposite were the results of the antiphlogistic plan, that Dr. Rasori, then a celebrated physician of Milan, began to entertain doubts of the most essential dogmas of Brown's doctrine; and though himself a zealous follower of it, and the first by whom the elementary work of that once celebrated man had been translated into Italian, with notes, yet he, after prudently substituting remedies of an antiphlogistic kind in the disease, obtained such favourable results, that he finally became convinced of the insufficiency of excitabilism.

On considering the circumstances which thus undeceived him, and observing the effects of several articles of the Materia Medica on the system, he professed the following positions, which, although not systematized in any particular work, are considered the rudiments by which the spirit of the new doctrine was disclosed to the world; namely,

1st. That the universal stimulant property of every thing on the system (which was inculcated by the Brownonians) was to be disproved; for there were substances that act with a contrary effect to that of stimulation, and which produce effects on the excitement, similar to what are produced by negative powers, as bleeding, purging, &c.

2nd. That by these substances, which have been called contra-stimulants, the effects of stimuli, or of increased excitement, are to be removed; and that when they are used beyond what is necessary, they are productive of bad effects, which can be removed by having recourse to the stimulant remedies again.

3rd. That, according to these premises, the morbid state, whatever it may be, that arises from the stimulant diathesis, is to be cured by contra-stimulants, as by bleeding, &c.; while, on the contrary, in *stimulants* we have a remedy for the contra-*stimulant diathesis*.

4th. That the living fibre bears doses of stimuli according to the prevalence of either diathesis.

5th. That this ability of the fibre is one of the most significant indications of the prevailing diathesis and its degrees, and more to be trusted to than the symptoms, which are fallacious and uncertain, often being common to both diatheses.

Such are the principles on which the New Medical Doctrine of Italy is founded. They never would, perhaps, have been divulged publicly to the world by Rasori, though, as one of the authors above specified says, they formed one of the most remarkable epochs in the history of medicine, had not Professor Tommasini, of Bologna, been their zealous advocate. By this eminent man, these rudiments have been systematized into a body of medical doctrine, and they have thence become rapidly diffused among the most celebrated schools of Italy.

The term *diathesis* is still retained; and, originating as this doctrine does from that of Brown, excitability is also held to exist, as that principle or property of life, on which the inherent powers of all animal organization depend. The doctrine being thus formed on the same basis, and being a reform of Brown's, has caused it to be styled the Daughter of Brownonianism.

Although it has been agitated among the schools for more than 20 years, it does not seem as yet to be wholly perfected. Its principal advocates, however, appear confident in the validity of its general principles, though there seems to exist some difference of opinion as to the classification of disease; and, in addition to the two diatheses, which are *stimulant* and *contra-stimulant,* some hold the existence of a *third,* to be termed the *irritative,* and which is to be cured without the use of either stimuli, or contra-stimuli, but simply by the removal of the cause of irritation. As to the importance of this, Tommasini is, however, not satisfied, for he considers the pathology as hinging on the simple division of disease into the stimulant and contra-stimulant diathesis.

This simplicity of classification has also been adopted in the Therapeia, and all remedies have been ranged under two grand classes, and consequently divided into *stimulants* and *contra-stimulants.* The principal ones belonging to the former are, opium, sulphur, æther, alcohol, camphor, and ammonia; while among the contra-stimulants are enumerated some noxious vegetables, as cicuta, aconitum napellus, atropa belladonna, linum palustre, lausocerasus, solanum, lactuca sylvestris, digitalis, nux vomica, and other vegetables, highly pernicious, whether indigenous or exotic, and even bitters, iron (which before were thought to be tonic), mercury, zinc, antimony, and other minerals, with the acids in general, all of which, opportunely administered, are held to produce directly the same effects which are indirectly produced by bleeding and purgatives, as before observed. As to *poisons,* the advocates of the new doctrine seem to hold, that there is no such thing in nature, if not used in an improper way.

Each class of disease has thus a class of remedies opposed to and adopted for their removal; and, as I noticed before, the ability of the system in bearing either class of remedies depends on the diathesis which prevails. So far is the basis of contra-stimulus founded on solidism, and the doctrine of Brown, in the upholding of excitability, and the simplicity of its dogmas. In its practical tendencies and general superstructure, it is, however, widely different; for, besides that, one of the idols of Brownonianism has been overthrown by the discovery of the contra-stimulant effect of *some* substances, another has shared the same fate, by the advocates of the Italian doctrine totally exploding the notion of *indirect debility*. Under the sway of this last idea, it was maintained by the Brownonians, that three diseases only in one hundred were of the sthenic diathesis. Now that such a dogma is laid aside, the supporters of contra-stimulants have severed the proportions, and hold that ninety-seven out of one hundred diseases come under the stimulant (or sthenic of Brown) diathesis; so that as the application of remedial means is affected, this is an important point of difference, and one which, while it manifests the superior merits of the new, no doubt, happily exposes the demerits of the old doctrine.

Contra-stimulus, though founded on excitability, "which," as one of the above writers said, "is a gratuitory idea," is thus not greatly at variance, in its practical application, with the more modern views of the tone of morbid action, which are entertained in England; for the proportion of the stimulant diseases equals that of the acute or phlogistic complaints, for which we employ antiphlogistic means, and the evacuating mode of treatment.

And although, in such a diathesis, contra-stimulants are liberally administered, positive depletion in many of them does not seem to be superseded, but is employed in the boldest manner. Whether or not the necessity of bleeding and purging is to be anticipated or prevented, in many diseases, by the administration of the contra-stimulant remedies of the materia medica, I am not sufficiently acquainted with the tenor and spirit of the new doctrine to say. In some, however, it appears probable, that the object may be obtained, by dispensing wholly with direct depletion; for Dr. Rasori himself was very successful in this manner of treating pulmonic affections; and, by gradually increasing the dose of the contra-stimulus, he sometimes gave up to 72 altogether of the tartar emetic, before the excitement yielded. In Naples, also, venesection is seldom (according to Valentin) employed in such complaints, but the cure is conducted by the use of tartar emetic, nitre, and digitalis.

The basis of this doctrine may be, as no doubt it is, like all that have preceded, subject to much controversy; in its practical indications it has much to support it, in the many corroborating modes of both ancient and modern treatment; while, in so ingeniously expressing the *modus operandi* of almost every reputed medicine and specific, by considering them all contra-stimulants, it seems to appropriate every concurring *datum* of the day to its own elevation, and, like Napoleon, it rises the child of circumstance. The honour of its discovery is, of course, justly claimed by the Italians; and though there does not seem to be any elementary work respecting it, that contains the full exposition of it in a system, its doctrinal points and facts occupy a good share of the Italian medical literature, and have no doubt carried their influence beyond the boundaries of the land that claims their birth. Whether or not our own country may have participated in its influence, I will not venture to say, from my long absence; but it is rather remarkable, however, that most of the important medicines of repute in modern practice are contra-stimulants in the language of the doctrine, and have been popular, posterior to the discovery of the Italian doctrine. Digitalis, thus, had its run, and has fallen back into the second class of remedial means in inflammatory complaints. Calomel has been but of late years the leading remedy in abdominal affections; while the tartrite of antimony, though considered always an important medicine, has, particularly of late, been directed to the removal of local and visceral inflammation.

In one sheet of paper, however, I shall not attempt to offer any further remarks on this subject, or to detail the observations that some Italian writers have made on it.

V.

*To the Editor of the Medico-Chirurgical Review.*

Sir,—As you informed me that you had already prepared for the forthcoming Number of your Journal an article on Labarraque's disinfecting liquids, which, under the various denominations of *chlorure—chlorides—chlorates*, and even *chlorurets*, of oxides of calcium and sodium, have been much talked of in France, and repeatedly announced in this country; I think it due to your readers and yourself to acquaint you, before that Number is published, that, by a series of chemical experiments which I have had the honour of reading before the Royal Society, at two of its meetings, within the last fortnight, I have proved, that the ideas entertained by the French author, as well as by all his commentators and translators, of the composition of those liquids, but particularly of that which contains soda, are perfectly erroneous; and that, therefore, the name already given to that liquid, as well as the one proposed to be given to it, by a recent writer on the subject, are inapplicable and incorrect. It is suprising that the happy and valuable application of those liquids imagined by Labarraque, should have been sent by him into the world without one word of scientific illustration or chemical analysis, but merely grounded on pure assumption; and that all his commentators and translators should have contented themselves with repeating and taking for granted what he had promulgated, without assuming the trouble of enquiring, practically, into the soundness of his views, before they became the heralds of his discovery.

1. Labarraque's liquid of soda does not contain a "chlorure d'oxide de sodium," (Anglicè, chloride of *soda*) but is a mixture of 73.53 chloride of *sodium*, (marine salt) and 26.47 chlorate of soda, in every 100 grains, with an excess of chlorine equal to twice the bulk of the water employed.

2. The salts contained in the liquid in question are *not* the disinfecting agents; for, on their being obtained in a dry state, by a gentle evaporation of the liquid, and again re-dissolved in distilled water, it is found that all the remarkable properties of the liquid are lost. Hence, the attempt to sell the pretended basis (or chloride of soda) of Labarraque's liquid, in a dry state, must be founded on ignorance, and must lead to disappointment.

3. The real disinfecting agent in the liquid is the chlorine which it holds in a free state, as laid down in No. 1; for, if to the solution of the dry salt in water, mentioned in No. 2, chlorine gas be added, equal to twice the bulk of the water, all the disinfecting properties are restored.

4. A similar quantity of chlorine gas, held in solution by distilled water, produces the same disinfecting results, and with equal rapidity, but with great inconvenience to the operator, owing to the escape of the gas, which excites cough, and causes a constriction of the trachea and headache—effects not observed in the case of the disinfecting liquid of soda. From this circumstance, it must be inferred that the presence of the salts serves to chain down, as it were, the excess of chlorine gas.

5. According to the atomic theory, Labarraque's liquid of soda consists of—

| | |
|---|---|
| 1 integrant atom of Chlorate of Soda | = 13.5. |
| 5 integrant atoms of Chloride of Sodium | = 37.5. |

6. The quantity of chlorine required to form these salts is considerable. Twenty fluid ounces of a solution of chrystallized carbonate of soda, prepared according to Labarraque's formula, absorb 503.36 cubic inches of chlorine gas, and form 725 grains of the mixed salts; besides which there are 69.43 cubic inches of the same gas remaining in a free state in the liquid. Of these facts Labarraque seems entirely ignorant.

7. The mode of preparing the liquid in question, proposed by that gentleman, is neither the easiest, nor the most economical.

8. The name which might, with propriety be substituted for the present erroneous one given to the liquid, is that of "disinfecting liquid of soda," and, when writing a Latin formula, that of "liquor chloro-sodaicus Labarraquii."

Having had occasion to use, more extensively, perhaps, than any other person in this country, the two disinfecting liquids of lime and soda, and made numerous experiments on dead bodies, putrid animal matter, putrid effluvia, &c. for the last 15 months, but more particularly during the whole of the last very hot summer, in the presence of a great number of gentlemen, I am enabled to state, in addition to the preceding information, that I have succeeded in ascertaining the *modus operandi* of chlorine on animal matter, in a state of putrefaction—that new compounds are formed during that action, which I have analyzed—that the presence of putrid effluvia in the air may be detected by chlorine—and that the easiest and most economical process of obtaining the liquid of soda for *disinfecting* purposes, is to saturate, with chlorine gas, a solution of muriate of soda, or common salt. These various points will be shortly developed, at full length, in a second communication to the Royal Society.

I have the honor to be, Sir,
Your very obedient Servant,
A. B. GRANVILLE, M.D. F.R.S.
*Physician in ordinary to H.R.H. the Duke of Clarence.*

*Grafton-st. Berkeley-sq.*

15*th March*, 1827.

---

VI.

### ADVERTISEMENT.*

THE following apology for BRITISH ANATOMY, and incitement to the cultivation of MORBID ANATOMY, would little avail, unless a plan for promoting both, but especially the latter, easy of execution, and admitting of extensive application in all the cities and principal towns of the empire, were submitted through the Profession to the Public, and its adoption invited by the example of its utility.

The plan was formed by DR. FARRE, in the autumn of 1825; was announced, and the Building for it commenced in the spring of 1826, and was more particularly detailed by him in a Course of Illustrations on Organic Diseases of the Heart and Aorta, which he gave to the Profession at the close of 1826. The ACADEMY for carrying it into effect will be opened for the admission of Members and Students, on the 26th of March, 1827.

---

*Plan of the Academy of Minute Anatomy, and Pharmaceutical Analysis.*

Firstly, To cultivate the Anatomy of Structure, as contradistinguished from the Anatomy of Relative Situation, which chiefly occupies the attention of the Schools.

Secondly, To give facility to post mortem examinations, for which a DEMONSTRATOR OF ANATOMY is provided, who will always hold himself at the service of those Medical Gentlemen who wish to encourage, but cannot command the time which is required for this pursuit.

Thirdly, To develop, by researches into Minute *Morbid* Anatomy, not only those organic changes which distinguish structural from functional diseases, but also to trace the peculiarities of the organization of morbid parts, which constitute the generic and specific characters in disease, and especially to observe the series of morbid appearances which occur successively in organs which are united, not only by the more obvious natural sympathies, but also by obscure morbid associations.

Fourthly, To publish, at stated periods, a JOURNAL OF MORBID ANATOMY, OPHTHALMIC MEDICINE AND SURGERY, AND PHARMACEUTICAL ANALYSIS, open to the Profession for the reception of communications, recorded on the responsibility of the contributors.

Fifthly, To publish, separately from the Journal, ESSAYS on the Subjects of the Illustrations in MORBID ANATOMY, occasionally given by DR. FARRE, with plain or coloured Plates, illustrating the successive changes of structure.

Sixthly, To cultivate the Fine Arts of DRAWING and MODELLING, as far as it is desirable that they should be connected with MINUTE PRACTICAL and MORBID ANATOMY. Most Anatomists have felt the disadvantage of being unacquainted with these Arts, and especially those who have more particularly directed their attention to Morbid Anatomy. The difficulty of preserving Morbid Appearances, and of obtaining an Artist to perpetuate them, renders this combination of studies very desirable. His friend, Sir Astley Cooper, distinguished as much by his zeal in this as in his other professional pursuits, and himself, to insure fidelity of representation in the execution of the Plates attached to their Works, retained a Miniature Painter in their joint service for several years, and educated him to the Profession.

---

* This Advertisement is intended to be annexed to the Abstract of an Introductory Lecture, which is preparing for Publication.

It is indeed highly desirable that all persons, who cultivate Medicine from a love of the science, should study painting, as far as it is subsidiary to the primary pursuit, for an accurate Sketch is a permanent Memorandum. He intends that every Student of the Academy, over whom he may have control or influence, shall both Dissect and Sketch.

This Academy is at present an instrument in the hands of an individual; it rests with the Medical Public to make it an Institution. Regarding it in its humblest form, he will consider his labour repaid, if it produce but one British Anatomist, deserving to rank with the eminent men who have preceded him.

As an instrument of Analysis, it will not interfere with the Schools of Anatomy, and its object being the Anatomy of Structure, which is chiefly applicable to the Physiology of Health and Disease, entire Subjects will not be admitted, although the Anatomy of Relative Position, as far as it is subservient to the primary pursuit, will not altogether be excluded. Demonstrations in a regular Series, on Select Subjects of Minute Anatomy in the Gastric or Absorbent, Cardiac, and Sexual, but more especially of the Cerebral Systems, and in particular, of the Eye, Ear, and other Senses, will be given by Mr. Dalrymple to the Pupils of the Academy, as opportunity may offer.

Pharmaceutical Analysis is blended with the pursuit of Minute Anatomy, with a view of obviating the difficulty often experienced by Medical Students of obtaining Pharmaceutical Knowledge. Many Physicians are defective in this part of their education. It is desirable that Students should have the opportunity, which they cannot now be said to possess, of gaining, by the readiest access to such Institutions, this knowledge, the importance of which to the Practitioner may be estimated, not only by the extent of its application to Medico-chirurgical Science, but also by its affording the only means of correcting, and finally putting down the adulteration to which drugs are so extensively subjected.

This part of the Academy is under the direction of Mr. Battley, whose liberal offer of erecting a Laboratory at his sole expense, for the instruction of the Students of the London Ophthalmic Infirmary, in his own Pharmaceutical Processes, made to the General Committee, shortly after the removal and pemanent establishment of that Institution in 1822, deserves, in this place, particular notice and acknowledgment.

A successive Analysis of the most important Articles of the Materia Medica will be conducted by Mr. Battley, in which the greatest pains will be taken to separate their efficient principles, as nearly as possible, in the state in which they exist in the drug, without permitting them to enter into new combinations. The elementary parts thus obtained, will then be recombined, and the difference between the new compound and the original drug illustrated.

Not only will the operations of the Pharmaceutical Laboratory, but also a Museum of Materia Medica, be open to the inspection of the Profession, and the study of the Pupils. The Museum will contain the finest Specimens of Galenical and Pharmaceutical Preparations, contrasted with those of an inferior kind. The different Processes of the various Pharmacopœias will be investigated, and the best means of obtaining them pointed out.

Every facility will be given to Anatomical and Phamaceutical Analysis, for the purpose of Medical Jurisprudence, and of Countervailing the Effects of Poisons.

Due notice of the Transactions of the Academy will be given in the Journal, at stated periods, and a Diary will always be open to its Visitors and Pupils.

Contributors of perfect Cases, that is, of accurate Histories, with careful Post Mortem Examinations of *all* the Cavities and External Condition of the Subject, with the Morbid Specimens, or of New Articles for the Materia Medica; or of Models, Drawings, or Instruction, to increase and diffuse Medical Science by aid of the Fine Arts, will be accounted *Honorary Members* of the Academy.

The Academy adjoins the London Ophthalmic Infirmary, has been erected, and is supported by the Saunderian Fund, which is independent of the Funds of the Infirmary. Voluntary Contributors to this Fund, either in or out of the Profession, will be accounted Benefactors, and constitute another class of *Honorary Members*.

Students who contribute to the Fund which supports the Academy, at the moderate rate which may be known at the Infirmary, will be considered as its Pupils.

*Notice of the Institution of an Annual Saunderian Lecture on Ophthalmic Medicine.*

It is intended that the First of these Lectures be given to the Profession, at The London Ophthalmic Infirmary, on *Wednesday*, the 28th of *March*, 1827, at *Four o'Clock* in the Afternoon, precisely, by Dr. Farre; and that the Morbid Anatomy of the Organ of Respiration be subsequently given, on *Monday*, the 28th of *May*, and on each succeeding *Monday*, at the same place and hour, until the subject be finished.

The *Card only*, of each Gentleman desirous of attending these Lectures, is required, which may be previously left at the Lecturer's House, *No.* 4, *Charterhouse Square.*

---

